# DRP1 mutations associated with EMPF1 encephalopathy perturb the transcriptional profile and maturation of cortical neurons

**DOI:** 10.1186/s11689-026-09713-0

**Published:** 2026-06-26

**Authors:** T. B. Baum, J. Costanzo, C. Bodnya, D. P. Boulton, M. C. Caino, D. Mogilenko, A. Zhelonkin, V. Gama

**Affiliations:** 1 Vanderbilt University, Cell and Developmental Biology and Vanderbilt Center for Stem Cell Biology, Nashville, TN USA; 2 Department of Pharmacology, University of Colorado Anschutz Medical Campus, Aurora, CO USA; 3 Vanderbilt University Medical Center, Nashville, TN USA; 4 Ben May Department for Cancer Research, University of Chicago, Chicago, IL USA

**Keywords:** DRP1, Mitochondria, Mitochondrial fission, Neurons

## Abstract

With the advent of exome sequencing, a growing number of children are being identified with de novo loss-of-function mutations in the dynamin 1-like (*DNM1L)* gene, which encodes the large GTPase essential for mitochondrial fission, dynamin-related protein 1 (DRP1). Mutations in DRP1 result in severe neurodevelopmental phenotypes, such as developmental delay, optic atrophy, and epileptic encephalopathies. Though it is established that mitochondrial fission is an essential precursor to the rapidly changing metabolic needs of the developing cortex, it is not understood how identified mutations in different domains of DRP1 uniquely disrupt cortical development and synaptic maturation. We leveraged the power of human induced pluripotent stem cells (iPSCs) harboring DRP1 mutations in either the GTPase or stalk domains to model early stages of cortical development in vitro. High-resolution time-lapse imaging of transport in neuronal projections revealed mutation-specific changes in mitochondrial motility of severely hyperfused mitochondrial structures. Transcriptional profiling of mutant DRP1 cortical neurons during maturation also implicated mutation-dependent alterations in synaptic development and gene expression of calcium-regulatory genes. Disruptions in calcium dynamics were confirmed using live functional recordings of 65–200 days in vitro (DIV) mutant DRP1 cortical neurons. These findings strongly suggest that altered mitochondrial morphology in DRP1 mutant neurons leads to pathogenic dysregulation of synaptic development and activity.

**Supplementary Information:**

The online version contains supplementary material available at https://doi.org/10.1186/s11689-026-09713-0.

## Introduction

Efficient, long-range transport of mitochondria is essential for proper cortical development [1–3]. This is evidenced by the fact that immature neurons have more highly motile mitochondria than fully mature neurons, to meet the metabolic needs of a rapidly changing cellular architecture [4]. Mitochondrial fission is a critical precursor to mitochondrial transport in order to travel through narrow axons [5–7]. The large GTPase dynamin-related protein 1, DRP1, is both a necessary and sufficient executor of mitochondrial fission [8–10]. Once mitochondria are transported to the distal, synaptic end of the cell, they join a local mitochondrial network where they can support fluctuating demands of ATP production and calcium buffering, allowing for proper synaptic function [11–13].

Advances in whole-exome sequencing have identified a growing number of patients with mutations in *DNM1L*, the gene encoding DRP1. These patients display severe neurodevelopmental phenotypes, including optic atrophy, developmental delay, microcephaly, and epileptic encephalopathies; most of these patients do not survive past childhood or early adolescence (Table 1) [14–17]. There is, therefore, a growing need to understand why early cortical development is particularly sensitive to disruptions in mitochondrial fission. Despite the severe, highly fused mitochondrial networks in patient cells [16], it is striking that individuals expressing these variants can support cortical networks during the beginning stages of fetal development and survive until birth. This study seeks to better understand how cortical neuron development and synaptic maturation are affected in DRP1-mutant cortical neuron cultures.

**Table 1:** Clinical Phenotypes of Patients with Variants in Mitochondrial Dynamics Genes

**Variant**	**Patient Phenotypes**	**Mitochondrial Morphology**	**PMIDs**
GDomain: E2A, G32A,S39N,S39G, T59N,W88fs, T115M, S36G/E116Kfs*6, W88Mfs*/E129K*6, D146N,G149R, A192E,K206N, K206E,G223V**, L230dup,R237W	optic atrophy, hypotonia, developmental delay, microcephaly, nystagmus, muscle tone abnormalities, ataxia, encephalopathy, mild hearing loss, seizures**	hyperfilamentous, elongated mitochondria, giant mitochondria, reduced mtDNA content	28969390 30085106 21701560 33387674 36212643 29110115 26825290 27328748 31868880 33718295 35914810 28667181
DNM1L (DRP1)			
Stalk A(middle): G359A,G359R, G362D,G362S, G362D/I512T,G363D, F370C,A395D**, A395G,G401S, R403C,E410K, C431Y	epileptic encephalopathy, developmental delay, microcephaly,pain insensitivity, mild speechdelay ataxia, muscle tone abnormalities, optic atrophy**	hyperfilamentous, elongated mitochondria, giant mitochondria, reduced mtDNA content, normal/abnormal peroxisome morphology	30801875, 31475481, 26992161, 26604000, 17460227, 30085106, 28667181, 30939602, 30109270, 35914810, 27145208, 31587467, 29877124
GEDDomain: Y691C,R710G, Q721Ter	epileptic encephalopathy, developmental delay, nystagmus, hypotonia	large, abnormal mitochondria and peroxisomes	30850373, 3591,810, 38341530

Patients with mutations in DRP1 display severe symptoms of neurodevelopmental regression, atrophy, and altered mitochondrial morphologies

^*^Mutation results in a stop codon. Symptoms reported in single patient, not reported in other patients with variants in this domain

**Fig.1:**
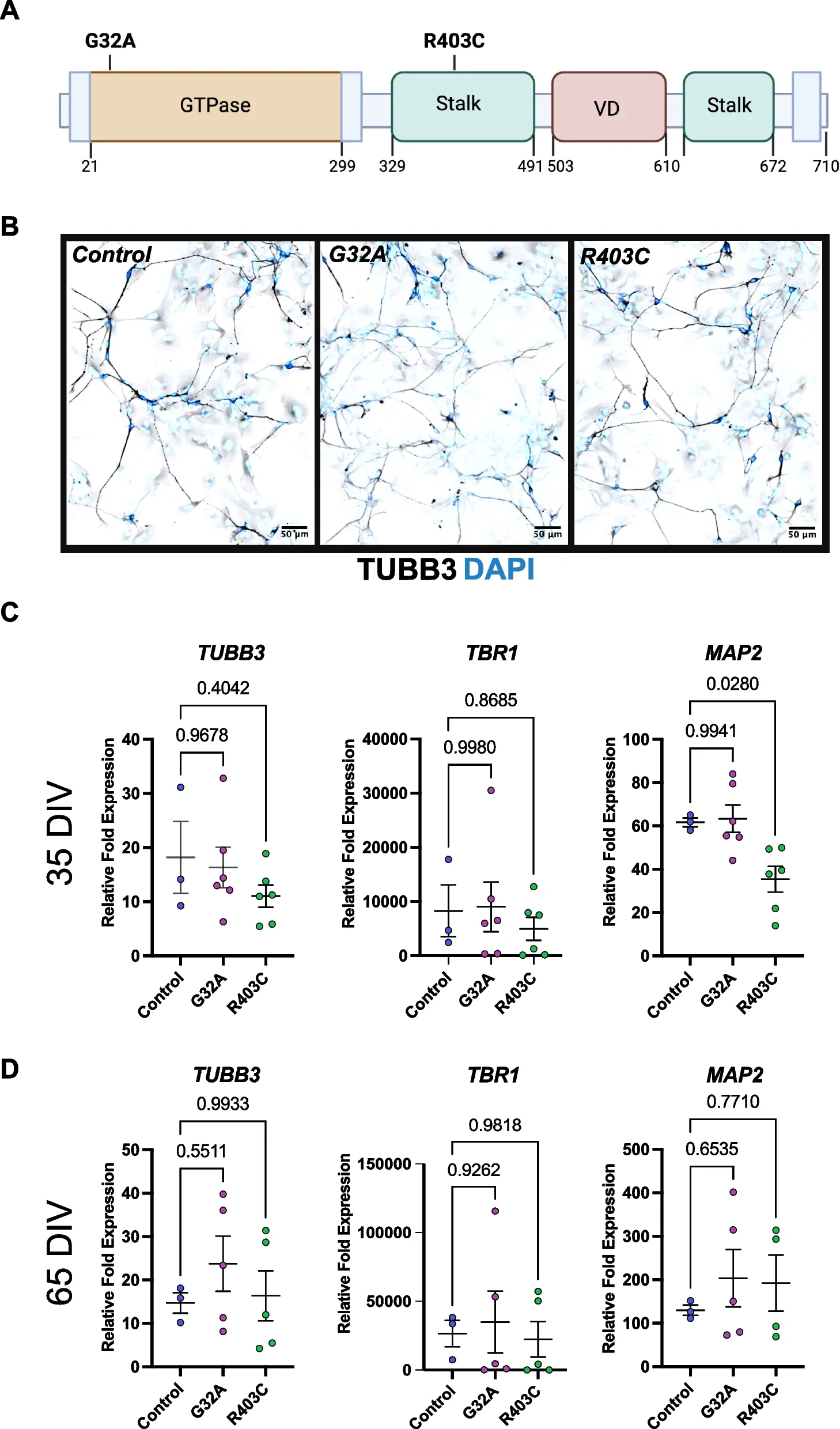
Characterization of mutant DRP1 cortical cultures. (**A**) Schematic of patient mutations in GTPase (G32A) or stalk (R403C) domain of DRP1 (**B**) Immunofluorescent staining of iPSC-derived 25 DIV cortical cultures for TUBB3/DAPI; scale, 50 µm (**C**) qPCR relative fold expression of markers of cortical differentiation *TUBB3, TBR1, MAP2* in mutant DRP1 cultures compared to control at 35 and 65 DIV (one-way ANOVA; control *n* = 3, mutant *n* = 5; 35 DIV MAP2 F2,10 = 1.32, df = 6.46, G32A *p* = 0.99, R403C *p* = 0.02; TUBB3 F_2,10_ = 0.43, df = 1.06, G32A *p* = 0.96, R403C *p* = 0.4; TBR1 F_2,10_ = 0.20, df = 0.35, G32A *p* = 0.99, R403C *p* = 0.86; 65 DIV MAP2 F2,10 = 1.33, df = 0.36, G32A *p* = 0.65, R403C *p* = 0.77; TUBB3 F_2,10_ = 0.1.74, df = 0.67, G32A *p* = 0.55, R403C *p* = 0.99; TBR1 F_2,10_ = 0.43, df = 0.15, G32A *p* = 0.92, R403C *p* = 0.98). Error bars represent mean ± SEM

In this study, we report the results of using human induced pluripotent stem cell (iPSC) lines harboring reported dominant-negative heterozygous mutations in either the GTPase or stalk domain of DRP1 (G32A or R403C, respectively) (Fig. 1A) to generate cortical cultures and characterize the effects of disrupted mitochondrial fission on neuronal development and synaptic maturation. We found that iPSCs with DRP1 mutations generate cultures with a similar composition of cortical neurons compared to controls. Mutant DRP1 neurons maintained highly elongated mitochondria within projections throughout maturation, and we subsequently observed significant size-dependent changes in mitochondrial motility that were mutation-specific. RNA-sequencing analysis of cortical neurons after 35 or 65 days in culture demonstrated mutation-specific differences in synaptic and other developmental genes that closely match the observed patient and in vitro phenotypes, providing new insights into the mechanisms underlying the changes we observed in neuronal activity.

## Results

### Differentiation of DRP1 mutant iPSCs into cortical neurons

DRP1 mutant iPSCs were differentiated into cortical neurons via small molecule dual-SMAD inhibition (Fig. 1B; Supplementary Fig. S1A) [18, 19]. Cortical neuron markers were quantified at 35 and 65 days in vitro (DIV) via qPCR (Fig. 1C-D). Transcriptional levels of *TUBB3* and *TBR1* were not significantly different between mutant DRP1 and control cultures at 35 DIV, indicating similar levels of glutamatergic cortical neurons in each culture. *MAP2* levels were found to be lower in DRP1 R403C mutant cultures compared to controls (Fig. 1C). By 65 DIV, transcriptional levels of *TUJ1, TBR1, and MAP2* in all mutant DRP1 neuronal cultures were comparable to controls (Fig. 1D).

### Hyper-elongation of mitochondria in DRP1 mutant cortical neurons

**Fig. 2:**
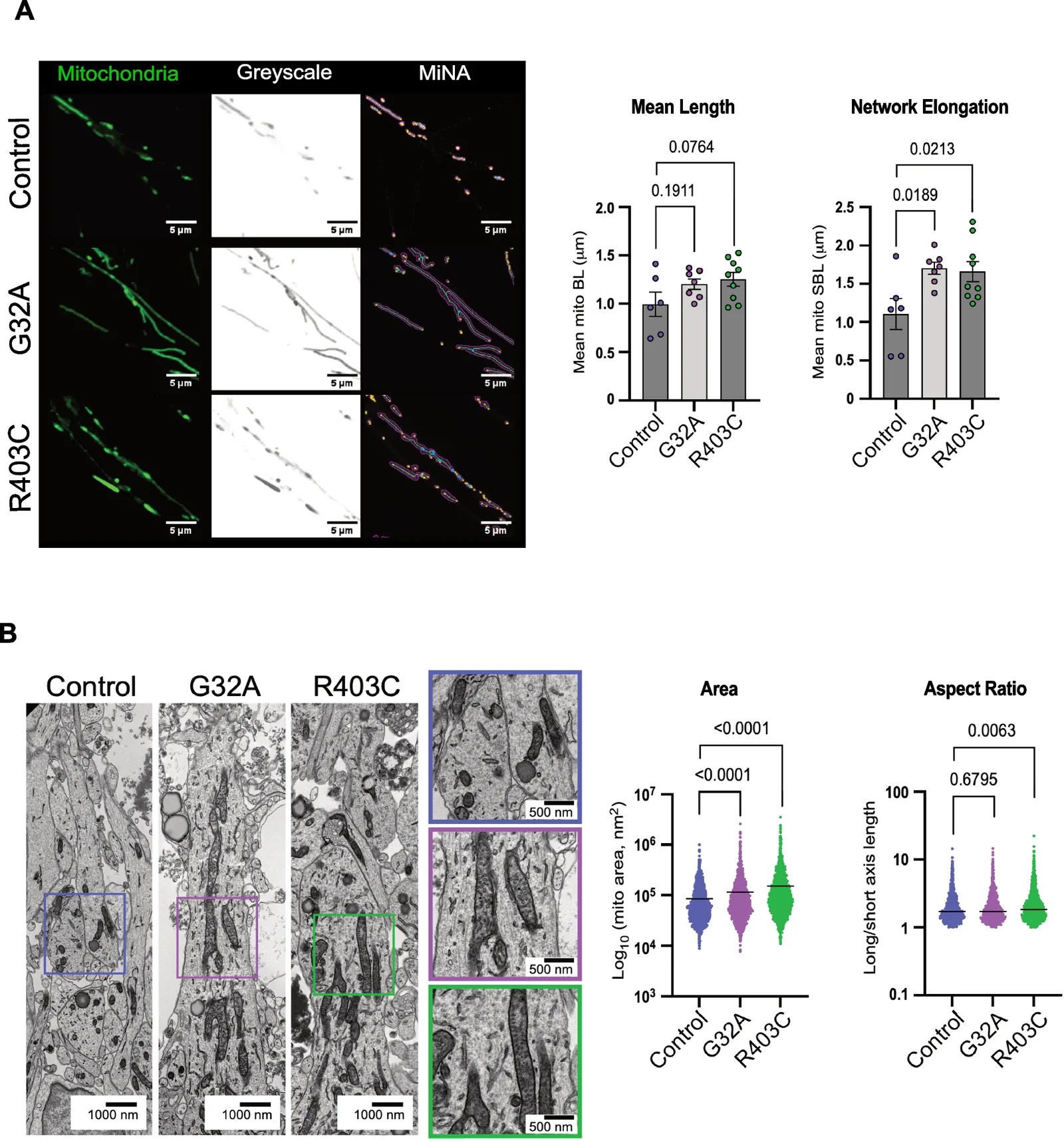
DRP1 mutations increase axonal mitochondrial size (**A**) SIM images of control or mutant DRP1 cortical neurons (G32A or R403C) stained with MitoTracker Green and analyzed via super-resolution microscopy and mitochondrial network analysis (MiNA). Left, representative images of mitochondrial morphology in neuronal projections are shown. Output MiNA annotated mitochondrial branches are shown. Right, Axonal mitochondria branch length (BL) and summed branch length (SBL) are represented as mean ± SEM and means were compared by one-way ANOVA and Dunnett’s post-test. Each dot represents the average from 2 clones and three independent biological replicates. (**B)** Mitochondria from cortical neurons expressing control or DRP1 mutations (G32A or R403C) were analyzed via transmission election microscopy imaging. Left, representative transmission electron images of gross mitochondrial morphology. Right, Axonal mitochondrial area and aspect ratio was quantitated and represented as individual dots (*n* = 1431–2183 mitochondria per group, including 2 clones in each group). Means were compared by one-way ANOVA and Dunnett’s post-test

Cells containing G32A or R403C mutations in DRP1 reveal a hyper-elongated mitochondrial phenotype resulting from an impaired capacity to undergo fission [16]. Because smaller, fragmented mitochondria are a critical precursor to efficient mitochondrial trafficking within neurons, it was unclear if hyper-elongated mitochondria would affect trafficking within neuronal projections. Control and mutant DRP1 cultures were fixed at 35 and 65 DIV and stained for TUBB3 and the mitochondrial marker HSP60. Mitochondria within neuronal projections were imaged at super-resolution and quantified for morphological differences between DRP1 mutant and control neurons. At 35 DIV, mitochondria that localized to neuronal projections in mutant DRP1 neurons had an increase in mean length and network elongation compared to control mitochondria (Fig. 2A). To complement these analyses, we performed transmission electron microscopy (TEM) and quantified the mitochondrial surface area and aspect ratio in neuronal projections. At 35 DIV, these morphological features were significantly increased in both G32A and R403C (Fig. 2B).

### Abnormal mitochondrial trafficking in DRP1 mutant cortical neurons

**Fig.3:**
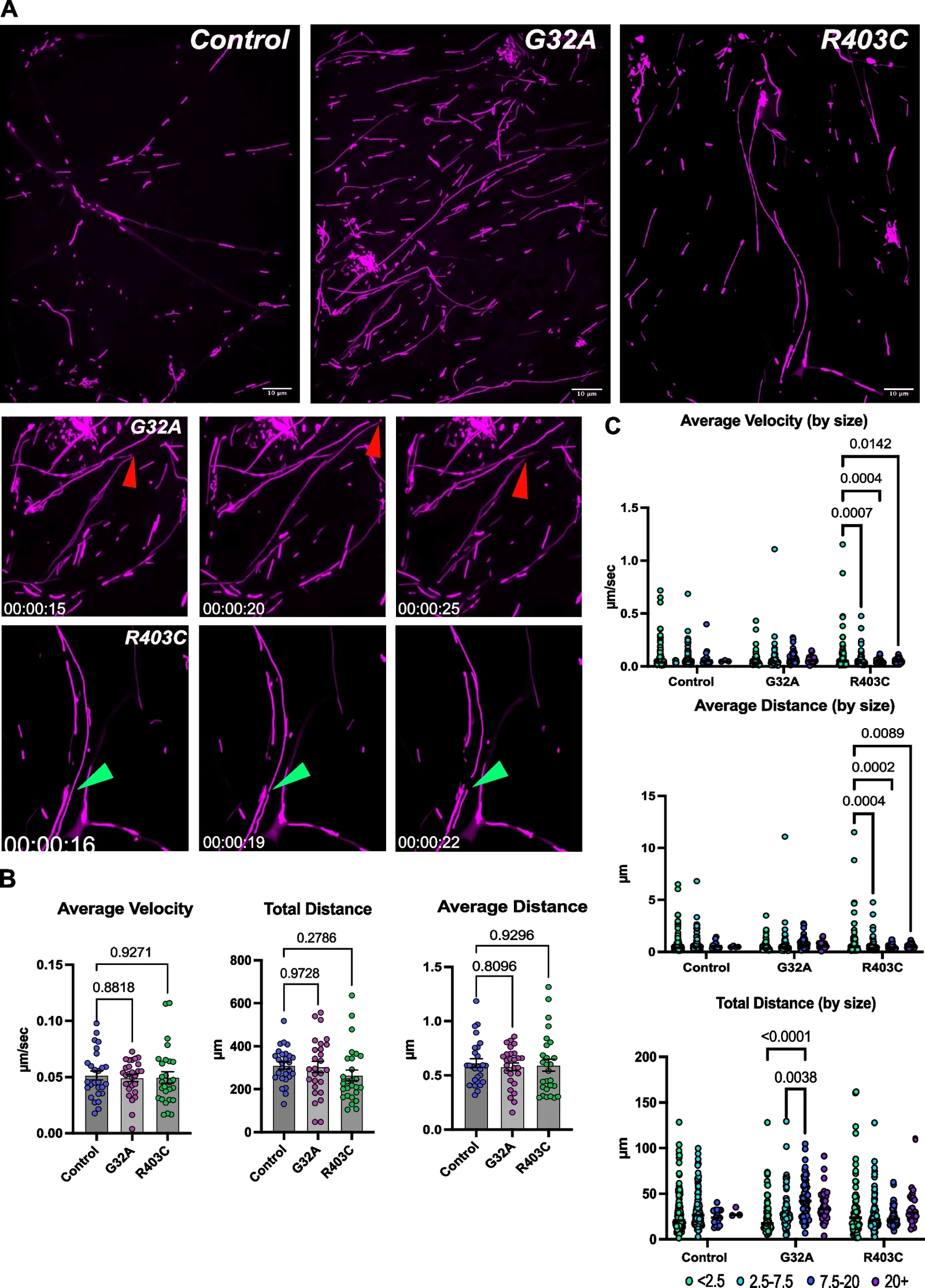
Motility of mitochondria in cortical projections (**A**) Spinning disk confocal max intensity projection of 65 DIV mitochondria (magenta) recorded for 10 min show dynamics such as extension and retraction (red arrow) and fission events (green arrow); scale, 10 µm (**B**) Average distance, average velocity, and total distance of all mitochondria movement averaged per projection (*n* = 30 projection, one-way ANOVA; average distance F_2,78_ = 1.26, df = 0.15, G32A *p* = 0.80, R403C *p* = 0.92; total distance F_2,78_ = 1.85, df = 1.16, G32A *p* = 0.97, R403C *p* = 0.27; average velocity F_2,78_ = 1.70, df = 0.09, G32A *p* = 0.88, R403C *p* = 0.92). Error bars represent mean ± SEM (**C**) Average velocity, average distance, and total distance of projection mitochondria movement binned by mitochondrial size (dots represent individual mitochondria, two-way ANOVA genotype x mitochondrial size, average distance F_6,736_ = 3.85, df = 6, *p* = 0.0009; total distance F_6,736_ = 5.137, df = 6, p < 0.0001; average velocity F_6,736_ = 3.85, df = 6, *p* = 0.0009; total distance F_6,736_ = 3.166, df = 6, *p* = 0.0045; p-values on graphs from Tukey’s multiple comparison post-hoc analysis). Bars represent median

Live recordings were acquired at 65 DIV for control and mutant DRP1 cultures to visualize the dynamic movement and trafficking of DRP1 mutant mitochondria within neuronal projections (Fig. 3). Overall, mutant DRP1 neurons at 35 and 65 DIV display a significant increase in projections containing hyper-elongated mitochondria compared to control neurons (Fig. 3A). Projections from mutant DRP1 neurons were observed to have many dynamic mitochondria undergoing both anterograde and retrograde movement, stalling, and rapid directional change, similar to control. Mitochondria within these projections were also observed to undergo some fusion and fission events (Fig. 3A, green arrow). Quantification of these events in neuronal projections is technically challenging; however, the mitochondrial event localizer (MEL) method [20] was used to estimate the number of fission events in 65 DIV R403C cortical neurons. Using this algorithm, it was confirmed that, in the 65 DIV R403C cultures, the number of fission events is significantly reduced, while the fusion events remain unchanged (Supplementary Fig. S1B-C, Video Set 1 Mitotracker).

Certain unique dynamic behaviors were observed frequently in mitochondria from mutant DRP1 neurons but were absent in control mitochondria, such as apparent extension and retraction of mitochondrial membranes on hyper-elongated networks without overall movement of the mitochondria (Fig. 3A, red arrow). Manual tracking of mitochondrial movement averaged across 10 projections per group showed no significant differences between mitochondrial movement distance (average and total) or average velocity (Fig. 3B) in mutant DRP1 and control neurons. Mitochondria in projections from mutant DRP1 neurons were observed to be highly heterogeneous in size, with possible size-dependent differences in motility. Mitochondria were therefore binned by size (small, < 2.5 µm; medium, 2.5–7.5 µm; large, 7.5–20 µm; very large, 20 + µm) and analyzed again for differences in movement distance and velocity. Interestingly, small mitochondria within projections in R403C mutant neurons moved a significantly further average distance and with a faster average velocity compared to medium, large, or very large mitochondria in the same projections (Fig. 3C). Large mitochondria within projections in G32A mutant DRP1 neurons were found to move a significantly further total distance compared to small and medium- sized mitochondria in the same projections. There were no significant differences in distance or velocity between mitochondria of different sizes in projections from control neurons (Fig. 3C).

### Transcriptomic profiling of DRP1 mutant cortical neurons

To define how DRP1 mutations alter cortical neuron development at the molecular level, we performed bulk RNA-seq on isogenic iPSC-derived cortical neurons carrying G32A or R403C DRP1 mutations at 35 and 65 DIV. Global quality control indicated a stable dataset suitable for downstream analysis. Sample-to-sample correlations based on log₂-CPM values were uniformly high (Pearson r > 0.85), with no obvious outliers (Supplementary Fig. S2A). Principal component analysis revealed that developmental timepoint was the primary source of transcriptional variation, with PC1 (35.1% of variance) clearly separating 35 DIV from 65 DIV samples, while PC2 (15.3%) captured somewhat heterogeneous genotype-associated differences within each timepoint (Supplementary Fig. S2B). This indicates that DRP1 mutations modulate, rather than overwhelm, the transcriptional program of neuronal maturation, motivating analyses that can detect both discrete mutation effects and developmental trajectory deviations.

**Fig. 4:**
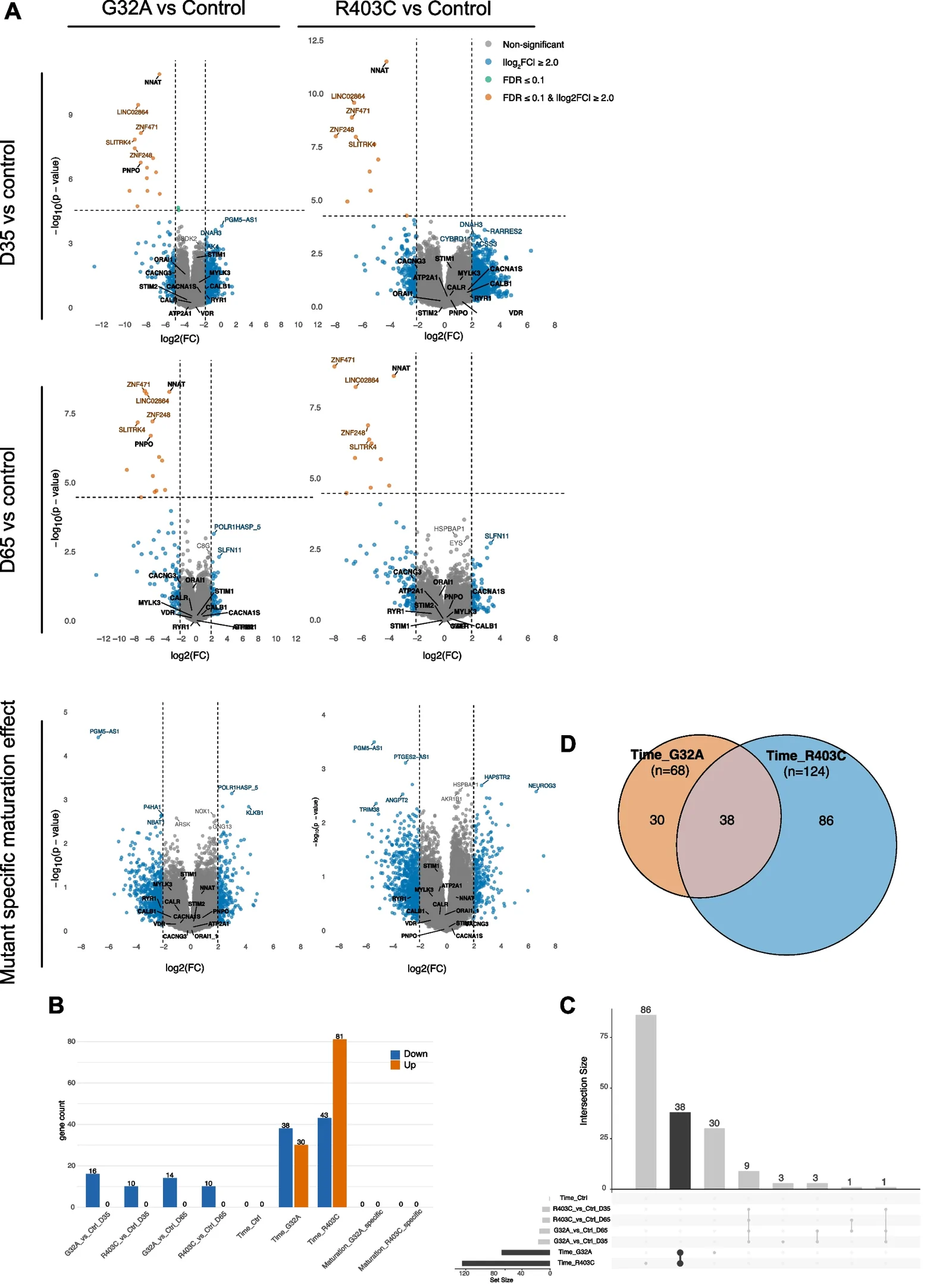
Differential gene expression in DRP1 mutant cortical neurons. (**A)** Volcano plots showing transcriptomic changes across developmental stages and genotypes. Top row: mutation effects at Day 35 (left: G32A vs Control; right: R403C vs Control). Middle row: mutation effects at Day 65 (left: G32A vs Control; right: R403C vs Control). Bottom row: mutation-specific maturation effects (left: G32A-specific developmental trajectory; right: R403C-specific developmental trajectory). Points are highlighted by significance (FDR < 0.1) with dashed horizontal line indicating FDR = 0.1 threshold. Y-axis shows − log₁₀(FDR); X-axis shows log₂(fold change) with vertical dashed line indicating log₂(fold change) ≥|2|. Calcium homeostasis genes are labeled with bold typeface. (**B) **Bar plot with the number of differentially expressed genes (DEGs) at FDR < 0.05 for each contrast (At FDR < 0.1 for the maturation mutant-specific contrasts no gene individually passes the threshold). Early mutation effects (D35) show 16 DEGs for G32A and 10 for R403C. Late mutation effects (D65) show 14 and 10 DEGs respectively. Maturation effects show 0 DEGs in control neurons, 68 in G32A, and 120 in R403C neurons. Mutant-specific maturation effects show 0 DEGs. (**C) **UpSet plot showing the intersection of significant DEGs across all contrasts. Set size bars (left) indicate the total number of DEGs in each contrast. Intersection bars (top) show the number of genes shared between specific contrast combinations. Connected dots below bars indicate which contrasts share genes in each intersection

We performed differential expression analysis using a factorial genotype × timepoint design with limma-voom and empirical Bayes moderation to assess both mutation effects at discrete stages and changes during maturation **(**Methods; Supplementary Table S1). At a stringent false discovery rate threshold (FDR < 0.05), mutation effects within each developmental stage identified relatively small sets of high-confidence differentially expressed genes (DEGs): at 35 DIV, 16 DEGs in G32A versus control and 10 DEGs in R403C versus control; at 65 DIV, 14 DEGs in G32A and 10 DEGs in R403C (Fig. 4A–B, Supplementary Fig. S3). Most of these genes showed large effect sizes (|log₂FC|≥ 2), and several were shared between the two mutations, consistent with partially convergent molecular responses despite affecting distinct DRP1 domains. Within-genotype maturation contrasts (D65 vs D35) revealed more extensive remodeling in mutant neurons than in controls. Control neurons showed no significant maturation-associated DEGs at FDR < 0.05, whereas G32A neurons exhibited 68 DEGs and R403C neurons showed 124 DEGs **(**Fig. 4A-B and Supplementary Fig. S2C). An UpSet plot (Fig. 4C) along with area-proportional Euler diagram of the two maturation contrasts [21] (Fig. 4D) showed that at FDR < 0.05 the mutant maturation responses share a distinct core of 38 genes (56% of the Time_G32A maturation DEGs, 31% of the Time_R403C maturation DEGs, Supplementary Table S2), while control neurons contributed no DEGs at FDR < 0.05 (at FDR < 0.1 Time_Ctrl have PIK3CD as the sole DEG, which intersects with the mutant maturation DEGs)**.** This pattern supports the interpretation that DRP1 mutations divert neurons onto altered transcriptional trajectories rather than simply accelerating or slowing a shared program. In contrast, interaction contrasts designed to detect gene-level mutation-specific maturation effects yielded no individual DEGs at FDR < 0.05 (Fig. 4B), suggesting that coordinated pathway-level changes might be more informative than single-gene tests and motivating the trajectory-based gene set enrichment analysis (GSEA) below.

### A trajectory framework for developmental pathway dynamics

To move from gene-level to system-level interpretations, we analyzed pathway enrichment across three contrasts that jointly describe each mutation’s evolution over time: Early maturation (35 DIV mutant vs control), TrajDev (mutation-specific deviation from the control maturation trajectory), and Late maturation (65 DIV mutant vs control).

**Fig.5:**
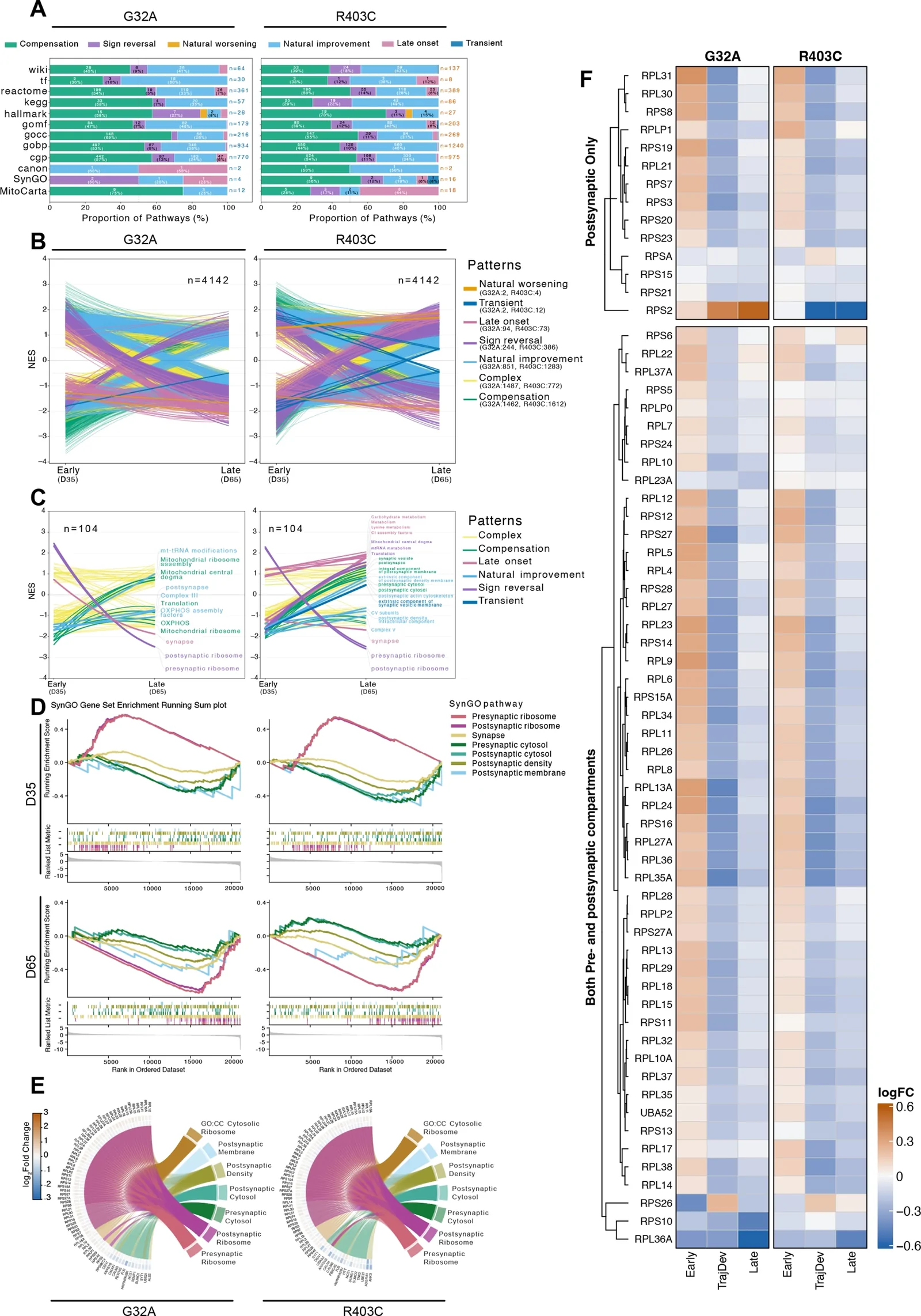
Developmental trajectory patterns reveal coordinated synaptic ribosome reversal in DRP1 mutant neurons. Gene set enrichment analysis (GSEA) was performed across 12 pathway databases (MitoCarta, SynGO, Hallmark, KEGG, Reactome, GO:BP/CC/MF, CGP, Canonical, TF, WikiPathways; > 17,000 gene sets total) for both G32A and R403C mutations. Pathways were classified into eight trajectory patterns based on their enrichment dynamics across three developmental contrasts: Early (35 DIV mutant vs control), TrajDev (mutation-specific deviation from control maturation trajectory), and Late (65 DIV mutant vs control). (**A)** Normalized pattern classification summary showing the distribution of trajectory patterns across all 12,221 tested pathways in G32A (left) and R403C (right) mutations. Each database is normalized to 100% to enable pattern proportion comparison. The Complex pattern (pathways not meeting criteria for the seven interpretable patterns) is excluded from visualization for readability and to focus on pathways with classifiable trajectory dynamics. Bar lengths represent non-Complex pathway counts per database; absolute counts shown as 'N (%)' inside bars; total non-Complex pathways per database shown at right as '*n* = X'. Pattern assignment is based on trajectory metrics (NES + FDR across Early, TrajDev, Late) for ALL pathways, not just those reaching significance thresholds. Patterns are ordered by mechanistic interpretation: Active patterns (Compensation, Sign reversal) require significant trajectory deviation (TrajDev FDR < 0.05, |NES|> 0.5); Passive patterns (Natural improvement, Natural worsening) show Early-to-Late changes without significant TrajDev; Other patterns include Late onset, Transient (Complex excluded for readability). (**B-C)** Bump charts showing trajectory dynamics of significantly enriched pathways across all databases (filtering out those classified as Complex in both genotypes to improve readability, *n* = 4,142). Each colored ribbon represents a pathway trajectory flowing from Early (D35) to Late (D65) for the 4,142 pathways where at least one mutation (G32A or R403C) exhibits a non-Complex pattern (i.e., meets criteria for Compensation, Sign_reversal, Natural_improvement, Natural_worsening, Late_onset, or Transient). Legend counts show pattern assignments per mutation; note that a pathway can have different pattern classifications in G32A vs. R403C (e.g., Complex in one mutation, Compensation in the other). Ribbon width is scaled by pattern frequency (rare patterns are thicker for visibility); vertical position indicates normalized enrichment score (NES) magnitude; color indicates trajectory pattern. *Curved lines* indicate pathways with significant trajectory deviation (TrajDev p.adjust < 0.05): the curve direction and magnitude are determined by the TrajDev NES value (mutation-specific maturation deviation), visualized using quadratic Bézier interpolation where the control point is displaced from the midline by `TrajDev_NES × curve_strength` (strength = 0.5). Importantly, the curves are introduced to facilitate pattern recognition, it is important to note though that on its own the presence of the curve does not nominate the pathway to be biologically significant. Positive TrajDev (upward curve) indicates pathway enrichment increases beyond normal maturation; negative TrajDev (downward curve) indicates enrichment decreases beyond normal maturation. Straight lines indicate pathways without significant trajectory deviation (passive patterns or insufficient statistical power). Left panel: G32A mutation; Right panel: R403C mutation. Divergent trajectories (ribbons crossing the horizontal zero line) represent pathways switching from upregulation to downregulation or vice versa (Sign reversal pattern). (**D)** GSEA running sum plots (enrichment plots) for top SynGO pathways at D35 (Early contrast, upper stack) and D65 (Late contrast, bottom stack). Each panel shows multiple GSEA enrichment curves for both G32A (blue) and R403C (orange) mutations. The running sum statistic (y-axis) reflects the degree to which genes in each pathway are over-represented at the top (positive enrichment) or bottom (negative enrichment) of the ranked gene list (x-axis: genes ranked by t-statistic). Vertical lines at the bottom indicate positions of pathway genes in the ranked list; peak deflection indicates normalized enrichment score (NES). At D35, synaptic ribosome pathways (presynaptic ribosome, postsynaptic ribosome) show strong positive enrichment (NES ≈ + 2.3–2.5, FDR < 1e-6), indicating ribosomal protein genes are concordantly upregulated in mutant neurons. By D65, these same pathways show equally strong negative enrichment (NES ≈ −2.5, FDR < 1e-7), representing a complete directional reversal in gene expression ranking. (**E)** Chord diagrams linking ribosomal protein genes to SynGO ribosome pathways for G32A (left) and R403C (right) mutations. Left side (genes): Each gene is represented by two stacked colored rectangles showing log2 fold-change (log2FC) at both timepoints—inner rectangle = D35; outer rectangle = D65; with colors on a blue-white-orange diverging scale (blue = downregulated, orange = upregulated). Gene names (RPL/RPS ribosomal protein families) are positioned radially outside the rectangles. Right side (pathways): Colored arcs represent SynGO synaptic ribosome and MSIGDB cytosolic ribosome pathways, SynGo synaptic membrane pathways. Ribbons: Curved connections from genes to pathways indicate leading-edge membership (genes contributing to pathway enrichment signal in GSEA); ribbon color matches pathway identity. Diagram demonstrates the gene set intersections between cytoplasmic ribosomal and synaptic ribosomal genes. Synaptic membrane and ribosomal genes, as well as cytosolic ribosomal genes show, though modest, yet coordinated downregulation from D65 to D35. (**F)** Expression heatmap showing temporal dynamics of SynGO-derived synaptic ribosome genes across the trajectory framework. Rows represent individual ribosomal protein genes (RPL/RPS family members) split by compartment annotation (Postsynaptic only vs Both compartments); columns show trajectory stages for two mutations: G32A (Early, TrajDev, Late) and R403C (Early, TrajDev, Late), six columns total. Color scale represents log2 fold-change (logFC) from differential expression analysis (blue = downregulation, white = no change, orange = upregulation; range −0.6 to + 0.6). Right annotation bar indicates SynGO compartment membership. The heatmap reveals coordinated gene expression patterns: modest upregulation at Early stage (orange in Early columns) transitioning through negative TrajDev slope (blue in TrajDev columns) to downregulation at Late stage (blue in Late columns), consistent with the pathway-level Sign reversal pattern. Notably, individual gene changes are modest in magnitude, and do not reach genome-wide significance (FDR < 0.05) when tested individually, but GSEA's ability to detect coordinated changes across gene sets reveals the highly significant pathway-level Sign reversal (pathway FDR < 1e-6)

GSEA across 12 pathway databases identified 5,267 gene sets enriched (FDR < 0.05) in at least one contrast, forming the primary analysis universe (Supplementary Table S3; Supplementary Fig. S4; Supplementary Fig. S5). Both mutations showed widespread pathway-level dysregulation, with the top interaction-contrast terms convergent between G32A and R403C (NES_TrajDev Pearson r = 0.94) (Supplementary Table S2). Each pathway was then assigned to one of eight mutually exclusive trajectory patterns**:** Compensation, Sign reversal, Progressive, Natural improvement, Natural worsening, Late onset, Transient, Complex. This assignment was based on the sign, magnitude, and significance of its Early, TrajDev, and Late NES values (Fig. 5A; Methods). Because GSEA contrasts cannot resolve which arm drives a trajectory change, pattern labels are complemented by per-replicate GSVA scores that distinguish active mutant dynamics from Ctrl developmental descent (Methods section f); the GSVA view is cited where the arm-level distinction matters for interpretation. Pattern labels are therefore descriptive of the interaction contrast, not of the replicate-level dynamics; the latter are reported in the GSVA driver classification.

The pattern distribution **(**Fig. 5A; full counts in Supplementary Table S3) revealed three key features. First, Compensation—active trajectory deviation opposing the initial defect—was the dominant active-response pattern in both mutations (G32A: 1,462/5,267 [28%]; R403C: 1,612/5,267 [31%]), accounting for 72% of pathways with a significant TrajDev in either mutation. Sign reversal—TrajDev-driven inversion of enrichment direction—was less frequent but present in both (G32A: 244/5,267 [5%]; R403C: 386/5,267 [7%]). Second, active amplification of initial defects (Progressive pattern) was entirely absent (0/5,267 in both mutations), and passive worsening (Natural worsening) was near-absent (≤ 4 pathways per mutation; specific pathways listed in Supplementary Table S2). Together, the near-complete absence of deteriorating trajectories indicates that neurons overwhelmingly mount adaptive rather than worsening transcriptional responses to DRP1 dysfunction. Third, the largest single category was Complex—pathways whose enrichment dynamics did not meet the shape criteria for any interpretable pattern (G32A: 52%; R403C: 41%)**;** a subset of these nonetheless carried significant TrajDev and represent active responses with atypically shaped trajectories (G32A: 11% of Complex; R403C: 10%). Per-pattern counts, definitions, and the full list of assigned pathways are provided in Supplementary Table S2. The 81-combination threshold-sensitivity analysis (Supplementary Table S4 and Supplementary Table S5) confirms these qualitative conclusions are stable: Compensation exceeds passive recovery in 81/81 combinations in both mutations, Progressive is absent in 81/81, and R403C carries more Compensation than G32A in 81/81.

To focus on pathways with active, mutation-driven trajectory changes, we next restricted the 5,267-pathway universe to those with a significant trajectory deviation component (TrajDev FDR < 0.05, |NES|> 0.5). This identified 2,039 pathways in G32A and 2,242 in R403C as active responders. Within this active-responder subset, Compensation was dominant (1,462/2,039 [72%] in G32A; 1,612/2,242 [72%] in R403C), and Sign reversal accounted for 244/2,039 (12%) in G32A and 386/2,242 (17%) in R403C. Notably, 291/2,039 G32A (14%) and 216/2,242 R403C (10%) active responders were classified as Complex, indicating maturation-specific responses that did not conform to the shape criteria of Compensation or Sign reversal. Late onset pathways (significant TrajDev without an Early defect) comprised the remainder (42/2,039 [2%] in G32A; 28/2,242 [1%] in R403C). Across all active-responder patterns, trajectories opposed rather than amplified initial defects**,** a distribution consistent with widespread adaptive neuronal responses (Fig. 5A).

### Visualizing trajectories across the interpretable subset

The bump chart traces each pathway's NES from Early to Late stage for the 4,142 pathways assigned a non-Complex pattern in at least one mutation (Fig. 5B). This set comprises 3,751 pathways that belong to the 5,267 ever-significant universe, and 391 that do not—these 391 fall short of FDR < 0.05 in every contrast yet attain trending significance (0.05 ≤ FDR < 0.10) in at least one mutation contrast, which is sufficient for the classifier to assign a non-Complex label (see Methods). We retain these 391 to avoid discarding pathways with biologically coherent trajectory shapes that narrowly miss the significance threshold. Curves in Fig. 5B mark pathways with a significant TrajDev component—i.e., Compensation and Sign reversal—and the Early and TrajDev NES of these pathways typically carry opposite signs, consistent with active responses that partially offset or fully reverse initial defects.

### A focused view on curated biological databases

To identify mechanistically interpretable trajectories within the unbiased 12-collection sweep, we restricted the focused visualization (Fig. 5C) to the 104 gene sets from two organelle-curated collections: mitochondrial (MitoCarta; n = 72) and synaptic (SynGO; n = 32), both of which had been loaded, size-filtered (10–500 genes), ranked, and FDR-corrected identically to the MSigDB collections (see Methods). The per-collection pattern distribution across all 12 databases is shown in Supplementary Fig. S5 and Supplementary Table S6.

Within the 12-collection sweep, we focused the visualization on two collections selected for different but complementary reasons: MitoCarta, on the biological prior that DRP1 controls mitochondrial fission and any DRP1-mutation effect must be read against the mitochondrial gene-set background; and SynGO, which surfaced from inspection of the interactive bump chart as the curated collection whose pathways occupied the universe extremes of |NES_TrajDev| — ranks #7, #17, #23, #49 of 5,267 (see Methods). The per-collection pattern distribution for all 12 databases is shown in Supplementary Fig. S5 and Supplementary Table S6.

These curated databases are included unconditionally, regardless of pattern classification, to ensure that all MitoCarta and SynGO pathways are visible for biological interpretation, even when their trajectory does not meet the strict criteria for an interpretable pattern label. Of the 104 pathways, 39 carry a non-Complex pattern in at least one mutation (and are therefore also present in the 4,142-pathway bump chart in Fig. 5B); the remaining 65 are classified Complex in both mutations and appear in the focused view (Fig. 5C) and in the interactive bump chart.

The two SynGO synaptic-ribosome pathways are the largest-amplitude signal in this focused view (Supplementary Fig. S6). Both presynaptic and postsynaptic ribosome trace a sign-reversing trajectory: strong positive enrichment at 35 DIV that flips to strong negative enrichment by 65 DIV**,** with the inversion captured by the trajectory-deviation contrast (postsynaptic ribosome TrajDev NES: G32A = −3.02, R403C = −2.89; presynaptic ribosome TrajDev NES: G32A = −2.90, R403C = −2.71; all FDR < 10–9). The deviations are near-identical between mutations and among the most extreme universe-wide—ranks #7 and #17 by |NES_TrajDev_G32A|, and #23 and #49 by |NES_TrajDev_R403C|, of the 5,267 ever-significant pathways. These two terms, however, do not annotate a molecularly distinct ribosome:

SynGO's presynaptic and postsynaptic ribosome terms annotate the same RPL/RPS proteins as the curated cytoplasmic ribosomal gene set — distinguished only by experimental evidence of synaptic localization [22]. All 70 SynGO synaptic-ribosome genes are members of the cytoplasmic ribosomal gene set (Jaccard = 0.574; the remaining 52 cytoplasmic genes are biogenesis factors and structural subunits not annotated as synaptic by SynGO). The SynGO "synaptic ribosome" is therefore not a molecularly distinct synaptic ribosome but the synaptically-localized fraction of the same structural ribosomal pool.

Turning from the synapse to the surrounding compartments (Fig. 4A; Supplementary Fig. S7) and the same biphasic inversion runs through the broader non-synaptic cytoplasmic structural-ribosome cluster — GO:CC cytosolic ribosome alone shows TrajDev NES = −2.56 (G32A) and −2.43 (R403C) — and through the cytoplasmic translation machinery as a whole, with KEGG translation initiation (TrajDev NES = −3.11/−2.96) and REACTOME eukaryotic translation elongation (−3.06/−2.95) among the strongest deviations recorded anywhere in the pathway universe (all FDR < 10–13). The synaptic-localized fraction sits at the amplitude extreme of this broader cluster, not as a phenomenon unto itself. The cytoplasmic ribosome-biogenesis machinery — assembly, processing, preribosome intermediates, nuclear export — runs in the opposite direction, a biphasic recovery classified as Compensation in both mutations (GO:BP ribosome biogenesis: G32A Early NES = −2.60, TrajDev NES = + 2.25; R403C Early NES = −1.72, TrajDev NES = + 1.59). At the contrast level, biogenesis is attempting compensation while the structural pool decompensates. The arm-level GSVA view (Supplementary Fig. S7) reveals what each Compensation label reflects at the replicate level—i.e., which arm is responsible for the trajectory change in the contrast. For the structural-pool modules — Synaptic Ribosomes and Cytoplasmic Translation — the inversion is a genuine crossover at the per-sample level: mutants fall from positive group-mean GSVA scores at 35 DIV to negative ones at 65 DIV while Ctrl samples themselves rise (Synaptic Ribosomes G32A: D35 mean = + 0.18 → D65 = −0.06, alongside Ctrl: D35 = −0.19 → D65 = + 0.21; Cytoplasmic Translation traces the same crossover). In the GSVA driver classification, these modules are labeled `both_moving` — reflecting a genuine per-sample crossover rather than a contrast artifact. For the biogenesis module, by contrast, the apparent gap-closure that GSEA flags as Compensation is driven by Ctrl developmental descent rather than mutant recovery (Ribosome_Biogenesis Ctrl: D35 = + 0.56 → D65 = −0.18; G32A: D35 = −0.28 → D65 = −0.15; R403C: D35 = −0.05 → D65 = −0.02). The biogenesis program does not, at the per-sample level, climb back up — Ctrl is coming down to meet it. The compensation attempt is real in the contrast view but registers as a closing gap rather than a rebound in the underlying samples. Accordingly, the biogenesis module is labeled `ctrl_driven` in both mutations. In the interactive dashboard, filtering by driver label lets the user isolate all `ctrl_driven` Compensation pathways across the full dataset and verify this pattern directly.

Against this backdrop, the mitochondrial gene programs themselves appear transcriptionally stable in the GSEA contrast view. The mitochondrial ribosome and its assembly machinery both classify as Compensation in both mutations and trace clean recovery arcs (Mitochondrial_ribosome: G32A Early NES = − 2.18, TrajDev NES = + 1.89; R403C Early NES = − 1.63, TrajDev NES = + 1.75; Mitochondrial_ribosome_assembly: G32A Early NES = − 1.99, TrajDev NES = + 2.11; R403C Early NES = − 1.89, TrajDev NES = + 1.86). The broader mitochondrial compensatory program — OXPHOS, mtDNA maintenance, Complex assembly factors — follows the same shape, and in R403C this Compensation signature extends across additional metabolic gene sets (lipid metabolism, amino-acid metabolism, ROS and glutathione metabolism, Complex V subunits, among others). The arm-level GSVA imposes the same Ctrl-driven caveat here as in the structural cluster: for Mitochondrial_Ribosome, Mito_Ribosome_Assembly, mtDNA_Maintenance and OXPHOS the apparent compensation is dominated by Ctrl descent (Mitochondrial_Ribosome Ctrl: D35 = + 0.32 → D65 = − 0.17; Mito_Ribosome_Assembly Ctrl: D35 = + 0.43 → D65 = − 0.30; mtDNA_Maintenance Ctrl: D35 = + 0.46 → D65 = − 0.26), with mutant samples staying flat or drifting further negative across the trajectory rather than climbing. The "DOWN-then-UP" arc in the contrast view is therefore better read as a flat mutant baseline relative to a declining Ctrl baseline.

In the GSVA driver classification (Supplementary Table 7), all four core mitochondrial Compensation modules are labelled `ctrl_driven` in both mutations. What remains robust across both views is the directional asymmetry between compartments: the cytoplasmic and synaptic structural-ribosome/translation programs undergo a true sign-reversing collapse at the per-sample level, while the mitochondrial programs invert—they remain at, or close to, the per-sample reference, and the apparent recovery in the contrast view is Ctrl descending to meet them. The organelle that the DRP1 mutation directly targets is, at the transcriptional level, neither failing in absolute terms nor visibly rescuing itself; the failure is in the cytoplasm and at the synapse.

Surprisingly, DRP1 acts on mitochondrial dynamics, yet the transcriptional collapse is not in the mitochondrial program, but in the cytoplasmic structural ribosome and translation machinery, and within that machinery, the synaptic-localized pool is the largest-amplitude. We hypothesize that a defect in mitochondrial distribution, rather than in mitochondrial intrinsic function, would energetically deprive synaptic compartments while leaving the mitochondrial population, taken as a whole, transcriptionally intact at the bulk level. The collapse of cytoplasmic and synaptic structural-ribosome/translation programs, against which a bulk-level biogenesis response is mounted but cannot restore the structural pool before the 65 DIV maturation window closes, is consistent with such a localized energy deficit at the synapse. Beyond the two ribosome terms, the SynGO compartmental landscape is mutation-asymmetric: in R403C, Compensation extends across both pre- and postsynaptic compartments (synaptic vesicle, pre- and post-synapse, their membranes and cytosol, the postsynaptic density and specialization membranes), while in G32A the matched pathways stay just below threshold — Complex in eight of nine cases, Natural_improvement in the ninth (postsynapse) — consistent with the broader G32A tendency toward subthreshold or atypically shaped active responses.

The interactive dashboard displaying all 5,323 pathways (the 5,267-pathway ever-significantly-enriched universe plus 56 curated MitoCarta/SynGO entries that did not reach ever-significant status) can be explored in any web browser. Each pathway entry includes a click-through GSVA panel showing per-replicate enrichment scores by genotype × timepoint, and a GSVA driver verdict indicating which arm is responsible for the observed trajectory change in the contrast. Sidebar filters allow the user to restrict the view by trajectory pattern, pathway database, NES range, or GSVA driver label.

This small number of coherent, biologically interpretable trajectories motivated focused analyses of synaptic ribosomes, mitochondrial metabolism, and calcium signaling.

### Synaptic ribosome trajectories suggest failure of sustained compensation

Per-contrast running-sum plots for both SynGO synaptic-ribosome terms, shown on a rank-normalized x-axis so the four contrasts are directly comparable, confirm that the directional reversal between 35 and 65 DIV is a broad shift in the enrichment statistic itself rather than a handful of leading-edge genes (Fig. 5D). The per-gene log2FC heatmap across the 65 expression-universe-filtered synaptic-ribosome genes traces the same arc — modest upregulation at D35, strongly negative TrajDev, pronounced downregulation at D65 — across both large and small subunits and across both mutations (Fig. 5F).

Chord diagrams place this signal in its wider compartmental context. The same RPL/RPS subunits that anchor the SynGO presynaptic and postsynaptic terms are also the dominant contributors to the non-synaptic cytoplasmic structural-ribosome pathways that share the Sign_reversal trajectory (Fig. 5E; Supplementary Fig. S7).The synaptic-localized genes are members of the broader cytoplasmic ribosomal pool; what looks at first like a synapse-specific failure is the amplitude extreme of a cytoplasmic-wide collapse.

### Mitochondrial pathways show partial transcriptional compensation

**Fig. 6:**
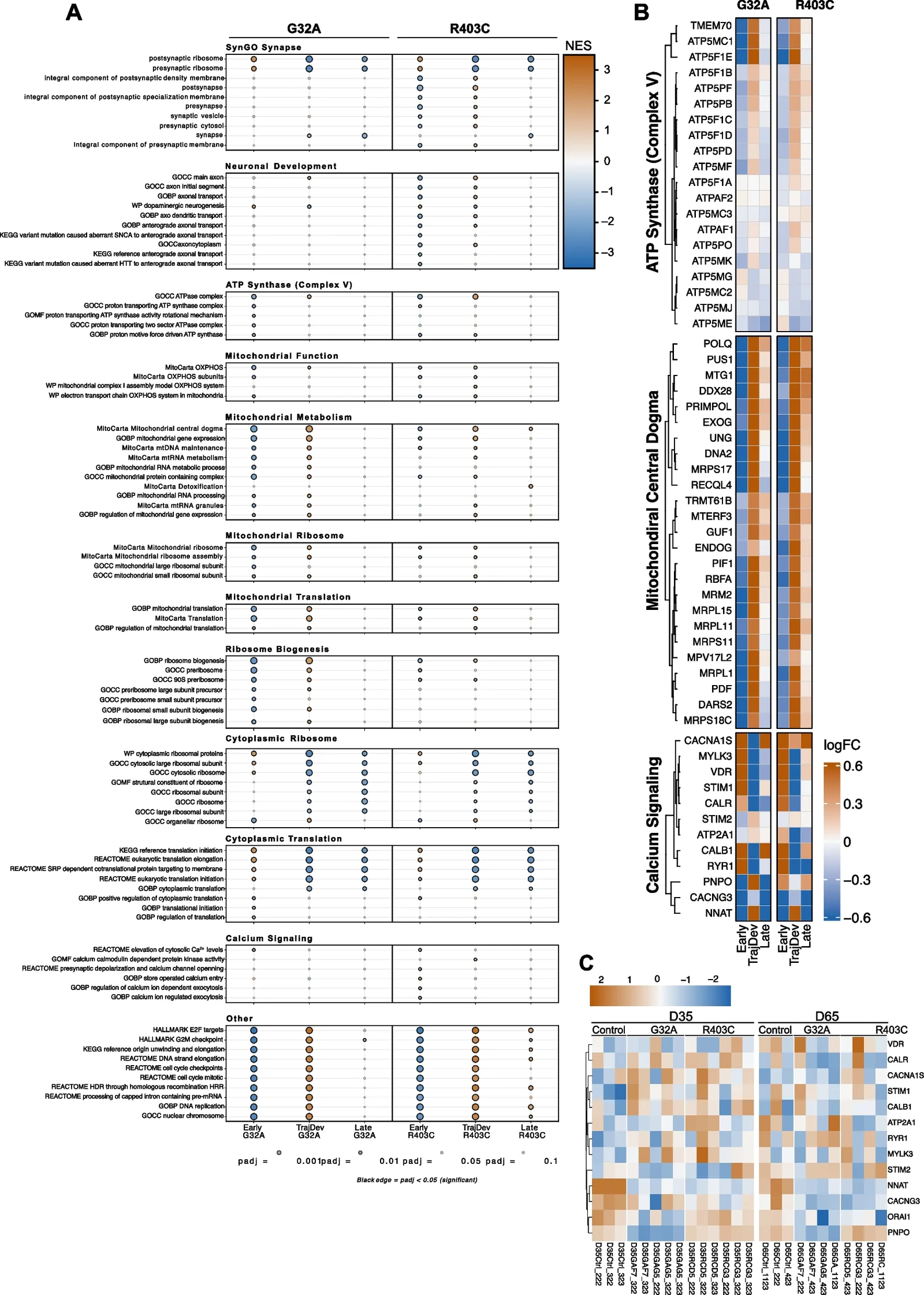
Divergent trajectories of mitochondrial and calcium signaling pathways reveal selective compensation in DRP1 mutant neurons. (**A)** Overview of curated pathway categories shows enrichment patterns across trajectory stages (Early, TrajDev, Late) for both G32A (orange) and R403C (blue) mutations. Dot color represents normalized enrichment score (NES); dot size reflects statistical significance. (**B)** Module-level trajectory heatmaps for three key biological systems: Top, ATP synthase (Complex V) genes demonstrate early suppression (D35: NES = −1.91 G32A, −1.89 R403C; both FDR < 0.02) followed by positive trajectory deviation (TrajDev: NES = + 1.49 G32A, + 1.68 R403C; FDR < 0.05) and partial recovery at D65, consistent with transcriptional compensation. Middle, Mitochondrial central dogma pathways (mitochondrial ribosome, ribosome assembly, mtDNA maintenance) exhibit similar compensation patterns with strong early deficits (NES < −1.6, FDR < 0.05) and significant opposing trajectory deviations (NES > + 1.7, FDR < 0.03). Bottom, Calcium signaling genes exhibit heterogeneous trajectory patterns with mixed directionality: some genes show early upregulation (CACNA1S, MYLK3, CALB1, RYR1), others early downregulation (NNAT, PNPO, CACNG3), with variable trajectory deviations that fail to achieve coordinated compensation. (**C) **Sample-level expression heatmap for curated calcium gene panel (*n* = 13 genes) reveals *NNAT* (neuronatin) as the most severely affected, with ~ 17-fold reductions in both mutations at D35 (G32A: logFC = −4.09, FDR = 2.7 × 10⁻⁷; R403C: logFC = −4.18, FDR = 6.4 × 10⁻⁸) that persist through D65. Heatmap colors represent log₂-transformed normalized expression values (logCPM); samples are grouped by genotype (Control, G32A, R403C) and timepoint (D35, D65)

Of 73 mitochondrial pathways significantly enriched at any stage (identified by "mitochondria", "OXPHOS", "respiratory", "electron transport" or "ATP synthase" across all databases plus MitoCarta origin), 23 of 27 G32A active responders (85.2%) and 13 of 32 R403C (40.6%) carry the Compensation label (Fig. 6A; Supplementary Figure S8; Supplementary Table S3 and Supplementary Table S8). At gene resolution, the mitochondrial Compensation signature is internally coherent. ATP synthase subunits (F1 catalytic, F0 channel, assembly factors) are coordinately depressed at 35 DIV and approach baseline by 65 DIV in both mutations (Fig. 6B); mtDNA-maintenance and mitochondrial-translation genes follow the same arc (Fig. 6B, middle). The `ctrl_driven` caveat established earlier applies to the whole set: gene-level coherence confirms the shape of the contrast-level recovery without changing the interpretation that Ctrl is descending to meet flat mutants.

### Calcium pathways resist pathway-level interpretation, but gene-level signals are coherent

Calcium-handling is the inverse case: noisy at the pathway level, clean at the gene level. Of 40 calcium-annotated pathways significantly enriched at any stage (identified by "calcium", "calci", or "ca2" in pathway description), only 1 G32A and 3 R403C neuronally plausible pathways (excluding cancer/tumor-derived signatures) carry an active Compensation label (Supplementary Table S3). Representative gene sets such as REACTOME ‘elevation of cytosolic Ca^2^⁺ levels’ are directionally ambiguous in the contrast view — early positive enrichment in both mutations (G32A NES = + 1.78, p.adj = 0.034; R403C NES = + 1.77, p.adj = 0.037) could reflect compensatory response, maladaptive upregulation of calcium-elevating genes, or indirect correlation, and GSEA cannot distinguish these.

Two genes carry the calcium signal as significant DEGs across the critical period are Neuronatin (NNAT) and pyridoxamine 5'-phosphate oxidase (PNPO). NNAT is a proteolipid that modulates ER calcium homeostasis by antagonizing SERCA, is the single most significantly dysregulated gene at 35 DIV in both mutations, with ~ 17–18-fold reductions (G32A: logFC = − 4.09, p.adj = 2.7 × 10⁻⁷, rank #1; R403C: logFC = − 4.18, p.adj = 6.4 × 10⁻⁸, rank #1), and the downregulation persists at 65 DIV (G32A: logFC = − 3.37, p.adj = 4.1 × 10⁻^5^, rank #3; R403C: logFC = − 3.59, p.adj = 2.5 × 10⁻^5^, rank #2). PNPO, essential for vitamin B6 synthesis and neurotransmitter production, is mutation-specific: persistently and significantly downregulated in G32A (D35: logFC = − 6.55, p.adj = 4.9 × 10⁻^4^; D65: logFC = − 5.77, p.adj = 6.9 × 10⁻^4^) but unchanged in R403C (D35: logFC = + 0.41, p.adj = 0.77; D65: logFC = + 0.34, p.adj = 1.0), consistent with broader metabolic consequences specific to GTPase-domain mutations.

Around these two anchors, the curated panel groups by expression module rather than by individual significance (Fig. 6C; Supplementary Fig. S2D). CACNG3 (calcium channel gamma-3, a TARP regulating AMPAR trafficking) and ORAI1 cluster with NNAT and PNPO by AveExpr and direction — both trend downward in mutant samples (CACNG3 ~ twofold in both mutations across both timepoints; ORAI1 modestly in G32A) — but neither passes the multiple-testing threshold (CACNG3 p.adj 0.45–1.0; ORAI1 p.adj 0.55–1.0), and ORAI1 is essentially null in R403C. Store-operated calcium entry components (STIM1, STIM2) and the buffering protein CALB1 do not follow the same pattern: STIM1 trends mildly upward, STIM2 is flat, and CALB1 trends upward at D35 in both mutations.

### Disrupted synaptic development in DRP1 mutant cortical neurons

**Fig. 7:**
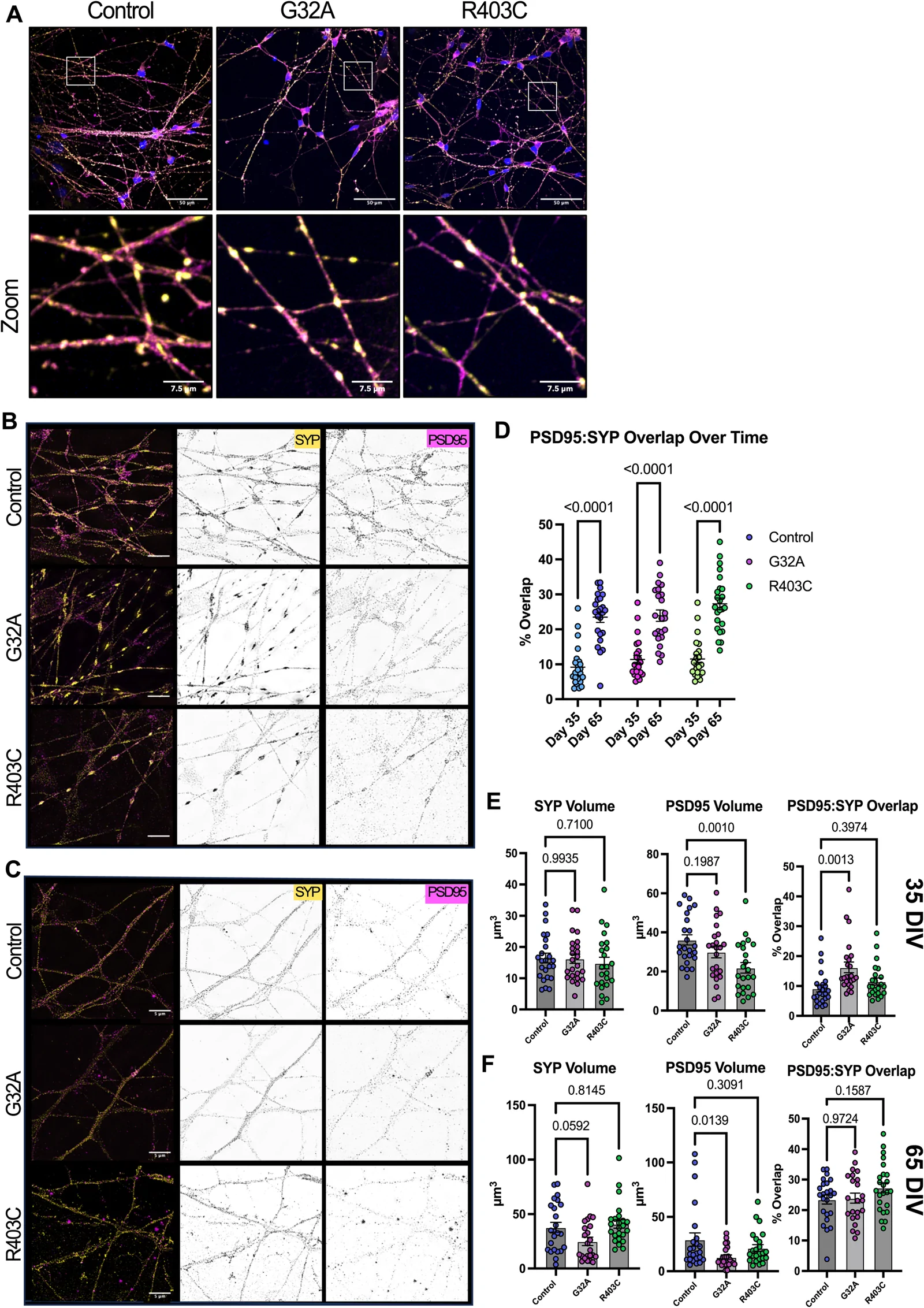
Synaptic development of mutant DRP1 neurons (**A**) Spinning-disk confocal max intensity projections of immunofluorescent staining for SYP (yellow) and PSD95 (magenta) in 65 DIV cortical cultures; scale, 50 µm. Zoom images: scale,   75 µm. (**B)** SIM max intensity projections of immunofluorescent staining for SYP (staining) and PSD95 (magenta) in 35 DIV cortical cultures; scale, 5 µm (**C**) SIM max intensity projections of immunofluorescent staining for SYP (staining) and PSD95 (magenta) in 65 DIV cortical cultures; scale, 5 µm (**D**) Quantification of change in % overlap of SYP and PSD95 between 35 and 65 DIV (paired t-test, *n* = 24 cells) (**E** and **F**) Quantification of synaptic density using SYP and PSD95 average total volume and colocalization in 35 and 65 DIV cultures (*n* = 24 cells; one-way ANOVA; 35 DIV total SYP volume F_2,68_ = 0.41, df = 0.28, G32A *p* = 0.99, R403C *p* = 0.71; total PSD95 volume F_2,68_ = 0.17, df = 6.6, G32A *p* = 0.19, R403C *p* = 0.001; colocalization F_2,68_ = 0.71, df = 6.6, G32A *p* = 0.001, R403C *p* = 0.39; 65 DIV total SYP volume F_2,68_ = 0., df = 0., G32A *p* = 0.059, R403C *p* = 0.81; total PSD95 volume F_2,68_ = 0., df =, G32A *p* = 0.01, R403C *p* = 0.3; colocalization F_2,68_ =, df =, G32A *p* = 0.97, R403C *p* = 0.15). Error bars represent mean ± SEM

The results of RNA-seq analysis indicate a significant change in genes related to synaptic development and activity in DRP1 mutant cortical neurons. During synaptic development pre- and post-synaptic structures develop proportionally to one another through activity-dependent positive feedback mechanisms [23–25]. To visualize differences in abundance and colocalization of pre- and post-synaptic proteins between mutant DRP1 and control neurons, cultures were fixed at 35 and 65 DIV and probed for pre-synaptic synaptophysin (SYP) and postsynaptic density protein 95 (PSD-95) markers using structured illumination microscopy (SIM) (Fig. 7). Visually, control DRP1 cultures appeared more synaptically dense, with a more complex network of interconnected neurites (Fig. 7A-C). Because pre- and post-synaptic structures develop concomitantly with each other, colocalization of SYP and PSD95 was quantified as a measure of overall synaptic strength [26]. A significant increase in overlap between SYP and PSD95 was found at G32A mutant synapses by 35 DIV compared to both control and R403C mutant synapses (Fig. 7B, E). By 65 DIV, there were no significant changes in the overlap of SYP and PSD95 at DRP1 mutant synapses compared to control (Fig. 7C, F). Overall, there was a notable increase in colocalization between SYP and PSD95 in all groups from 35 to 65 DIV (~ 10–15% to ~ 25–30% mean overlap), suggesting synaptic strengthening and network development over time in all cultures (Fig. 7D). To identify non-specific colocalization (density effects) we rotated the images of one of the markers by 90° and 180° and repeated the analysis [27] (Supplementary Fig. S9). This colocalization is presented as the percentage of PSD95 that overlaps with the synaptophysin signal and shows no significant overlap.

### Altered calcium signaling activity of DRP1 mutant cortical neurons

Intracellular calcium, one of the most common second messengers in the brain, is tightly regulated in neurons as an important mediator of cell proliferation, death, and synaptic plasticity [28, 29]. As action potentials arrive at the synapse, the activation of voltage-gated Ca^2+^ channels dictates the amount and duration of neurotransmitter release; mitochondria are essential regulators of intracellular Ca^2+^ ion compartmentalization, providing a buffer that maintains high sensitivity to Ca^2+^ fluctuations at synapses [30, 31]. Intracellular calcium, one of the most common second messengers in the brain, is tightly regulated in neurons as an important mediator of cell proliferation, death, and synaptic plasticity [28, 29]. As action potentials arrive at the synapse, the activation of voltage-gated Ca^2+^ channels dictates the amount and duration of neurotransmitter release; mitochondria are essential regulators of intracellular Ca^2+^ ion compartmentalization, providing a buffer that maintains high sensitivity to Ca^2+^ fluctuations at synapses [30, 31]. According to RNA-seq results, both G32A and R403C mutant DRP1 cultures show a decrease in expression of *CACNG3* compared to control cultures at 65 DIV (Fig. 6 and Supplementary Fig S2). *CACNG3* is an AMPA receptor regulatory protein involved in the modulation of trafficking and gating of AMPARs at the plasma membrane [32]. At both 35 DIV and 65 DIV, the proteolipid *NNAT* was the most significantly altered DEG in both R403C and G32A mutants compared to control, respectively [33, 34]. *NNAT* was initially discovered in rat brain tissue, where it was shown to regulate the intracellular Ca^2+^ signals necessary for brain development [35–37]. The protein levels of NNAT were significantly decreased in both G32A and R403C mutant DRP1 cultures compared to the control (Supplementary Fig. S10).

**Fig. 8:**
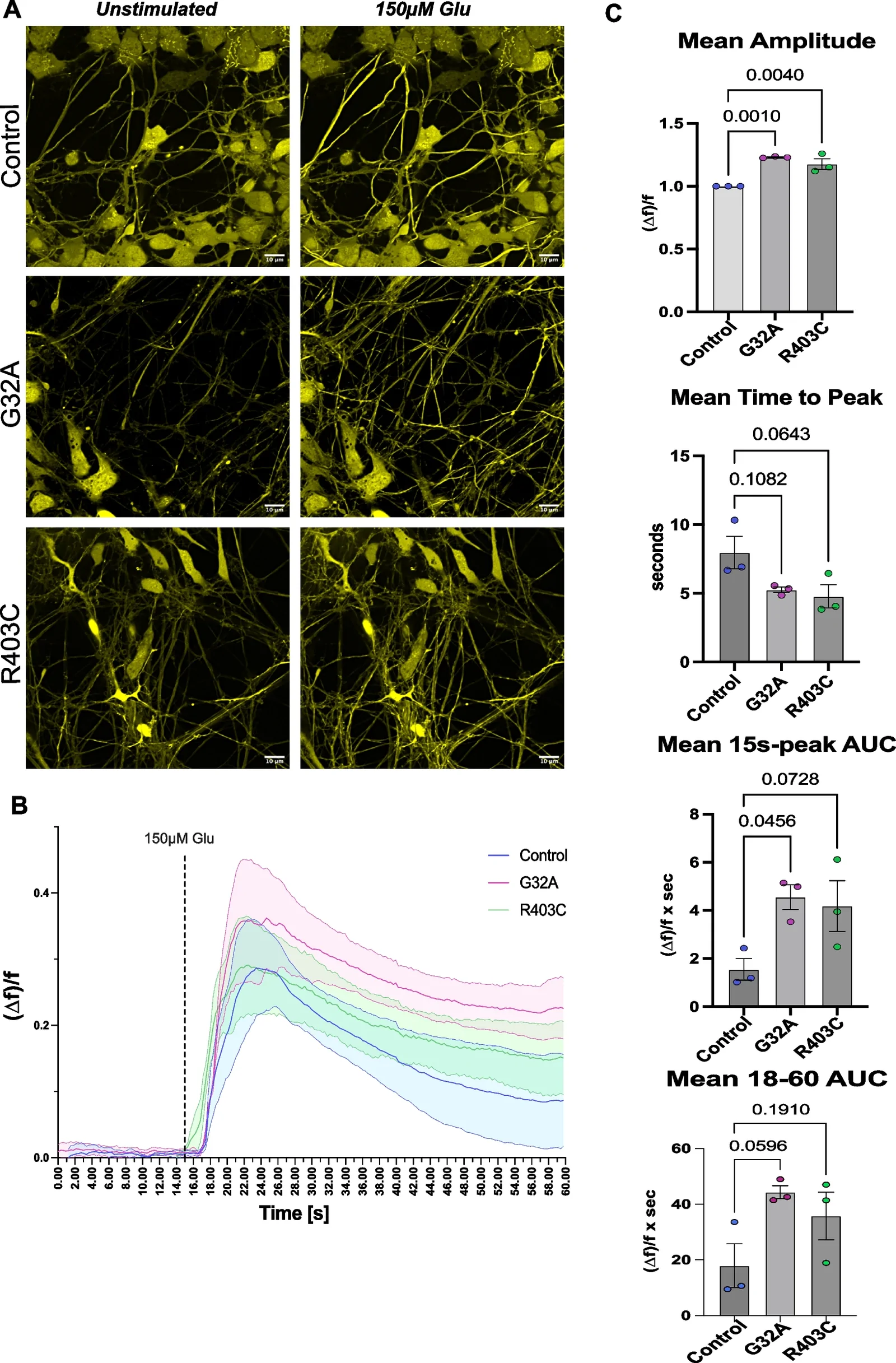
Calcium Response in Mutant DRP1 Cultures (**A**) Calcium response (Fluo-4AM, yellow) in confocal single-plane images of 65 DIV cortical cultures recorded for 1 min before (left) and after (right) 150 µM Glu stimulation in control and mutant DRP1 cells; scale, 10 µm (**B**) Change in fluorescence traces over time (average response across 50 ROIs per replicate) pre- and post Glu stimulation at 15 s in control and mutant DRP1 cultures (**C**) Mean amplitude of change in fluorescence, mean time to peak after Glu stimulation, and area under the curve from Glu stimulation to peak response (individual ROIs normalized to control per replicate, one-way ANOVA; mean amplitude F_2,6_ = 1.85, df = 24.23, G32A *p* = 0.001, R403C *p* = 0.004; mean time to peak F_2,6_ = 0.41, df = 4.14, G32A *p* = 0.10, R403C *p* = 0.06; mean 15 s to peak AUC F_2,6_ = 0.65, df = 5.12, G32A *p* = 0.04, R403C *p* = 0.07; mean 18–60 AUC F_2,6_ = 3.87, df = 0.38, G32A *p* = 0.059, R403C *p* = 0.19) of control and mutant DRP1 calcium traces. Error bars represent mean ± SEM

These results, as well as changes in pre- and post-synaptic markers, indicate a strong likelihood of perturbation of calcium dynamics in mutant DRP1 neurons. To visualize potential changes in calcium dynamics, 65 DIV cortical cultures were treated with cell-permeant calcium dye Fluo-4 AM and imaged immediately using high-resolution confocal microscopy. Cells were cultured in artificial cerebrospinal fluid (aCSF) media and recorded briefly to establish baseline activity, followed by subsequent treatment with 150 µM of glutamate (Glu) to stimulate neuronal activity (Fig. 8) [38]. Cultures were found to be spontaneously active at baseline without addition of agonist (Supplementary Fig. S11, Video Set 2 Calcium Signaling), and highly synchronously reactive post Glu addition (Fig. 8A). Mean fluorescence over time was calculated by averaging across 50 neuronal projection ROIs both at baseline and after Glu stimulation per replicate (Fig. 8B). Mean amplitude of change in fluorescence after Glu stimulation was significantly higher in both G32A and R403C mutant groups, indicating a larger Ca^2+^ response compared to control (Fig. 8C). Mean time from stimulation to peak response trended lower for both mutant DRP1 cultures, with R403C approaching a significantly lower time compared to control (Fig. 8C). Area under the curve was significantly higher in G32A DRP1 cultures and approaching significantly higher in R403C DRP1 cultures compared to control (Fig. 8C).

**Fig. 9:**
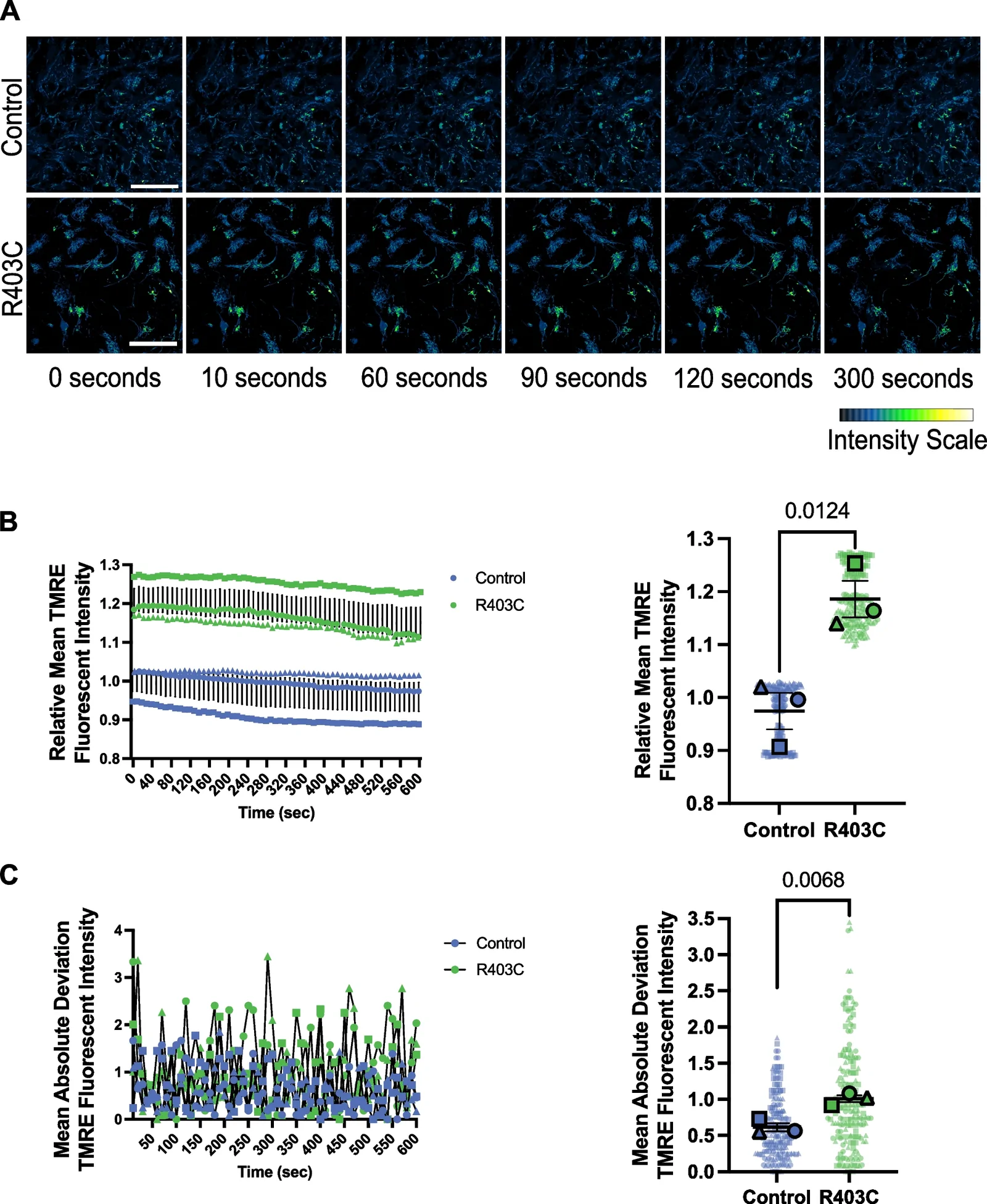
Changes in Mitochondrial Membrane Potential. (**A**) Spinning disk confocal max intensity projection of 60 DIV cortical neurons from biological replicates (*n* = 3) of control and R403C. Neurons were labeled with 500 nM TMRE for 10 min and 200 nM MitoTracker Green for 30 min at 37 °C. Live-cell imaging was performed using spinning disk confocal microscopy, with images acquired every 10 s for 10 min using the 100 × objective; scale, 40 µm (TMRE = green blue fire). (**B**) Graph of the relative mean fluorescent intensity of TMRE plotted against time (left panel), and quantification of the relative mean fluorescent intensity of TMRE (right panel) (*n* = 3; unpaired t-test with Welch’s correction; t = 4.329, df = 4.000, *p* = 0.0124). Error bars represent mean ± SEM. (**C**) Graph of the relative mean absolute deviation of TMRE fluorescent intensity plotted against time (left panel), and quantification of the relative mean absolute deviation of TMRE fluorescent intensity (right panel) (*n* = 3; unpaired t-test with Welch’s correction; t = 5.272, df = 3.871, *p* = 0.0068; Rout’s Outliers test, Q = 1%, control outliers = 2.31, 2.25; R403C outlier = 6.98). Error bars represent mean ± SEM. Relative values were background corrected and normalized to the mean of controls at time 0

To correlate changes in calcium signaling with mitochondrial activity, we measured mitochondrial membrane potential and detected a significant increase in the mean TMRE intensity and the mean absolute deviation, indicating flickering over time in mutant DRP1 cultures (Fig. 9, Video Set 3 TMRE). These findings indicate a more robust mitochondrial activity in mutant DRP1 cultures compared to control at 65 DIV.

The rate-limiting enzyme PNPO is required for the synthesis of pyridoxal-5’-phosphate (PLP), the active form of vitamin B6 and a critical enzymatic cofactor [39, 40]. As a vital cofactor, PLP is required for neurotransmitter synthesis, including the conversion of glutamate to gamma-aminobutyric acid (GABA). Moderate reductions in PLP levels have been shown to induce neuronal hyperexcitability and an increase in the frequency and intensity of calcium signaling (PMID: 40,625,348). Mutations in *PNPO* are known to be causal for an autosomal recessive pyroxidal-5’-phosphate (PLP) vitamin-responsive epileptic encephalopathy leading to refractory seizures during the first year of life [41, 42]. Therefore, PNPO deficiency and some mitochondrial encephalomyopathy, lactic acidosis, and stroke-like episodes (MELAS) patients may have convergence of pathways related to disease etiology and possible therapeutic overlap. Western blot was used to confirm whether transcriptional changes in the top DEX genes, including *PNPO* and *PCDHGC3*, persist at the protein level in mutant DRP1 cultures at later time points (over 100 DIV). Protein levels of PNPO and PCDHGC3, a calcium dependent cell adhesion protein, were found to be significantly decreased in G32A mutant DRP1 cultures compared to control, with little to no protein levels detected (Supplementary Fig. S10). Overall, these findings recapitulate changes in gene expression detected via RNA-seq profiling at 35 and 65 DIV and confirm their persistence as mutant DRP1 cultures continue to mature past 100 days in culture.

## Discussion

It is well established that highly dynamic mitochondria are essential for proper neurodevelopment, with multiple studies examining the complex quality control mechanisms that ensure the proper distribution and movement of mitochondria within axons and dendrites [43–45]. It is therefore of interest to understand how neurons with fission defects can survive during early stages of neurodevelopment. In this study, high and super-resolution imaging of neurons with mutant DRP1 reveals the capacity for highly elongated mitochondria to be actively trafficked within neuronal projections and to exhibit some dynamic behaviors, such as elongation and fragmentation of the network. Rapid directional movement, both anterograde and retrograde, indicates that these elongated mitochondria are actively trafficked, likely via the canonical axonal motor proteins kinesin and dynein on microtubule highways. Elongated mutant DRP1 mitochondria were also observed to extend and retract in a stretching motion, without lateral movement of the whole mitochondria. This motility behavior could be linked to an attempt and failure of microtubule motors to transport some very large mitochondrial cargo. Studies have shown that neurons have quality control mechanisms that help regulate the size of mitochondria traveling within axons and dendrites [4, 46]. As it is currently unclear at what size mitochondria can no longer be transported, these fission-defective cells provide a new opportunity to study the transport of large cargo within neuronal projections. Mitochondria in mutant DRP1 neuronal cultures were found to be highly variable in size, with significantly increased numbers of large (7.5–20 µm) and very large mitochondria (20 + µm) compared to control projection; R403C mitochondria were significantly more elongated compared to control mitochondria. Live recordings indicated that changes in mitochondria motility are size-dependent, with small R403C mitochondria moving further and faster on average than any other larger mitochondria within the same projection. Additionally, large G32A mitochondria moved further overall compared to smaller mitochondria within the same projection. These mutation-specific differences in mitochondrial motility (which are absent in control projections) indicate a possible compensatory response. Mutant DRP1 neurons may leverage their highly variable mitochondrial size caused by fission disruption to increase mitochondrial transport and ultimately cell survival. It is possible that disruption of proper mitochondrial motility interferes with the effective transport of other cargoes within the projection, further hindering necessary cell components from reaching the synapse. More studies are needed to support this possibility.

RNA-seq analyses of neurons at 35 and 65 DIV revealed substantial transcriptional differences between DRP1 mutant and control cultures, with the strongest and most internally consistent trajectory signal arising from the cytoplasmic translation machinery. At 35 DIV, the SynGO presynaptic and postsynaptic ribosome gene sets were positively enriched in both G32A and R403C neurons; by 65 DIV both had reversed to strongly negative enrichment, with trajectory-deviation magnitudes ranking among the most extreme observed across the 5,267-pathway universe (TrajDev NES ≈ − 2.9 to − 3.0; FDR < 10⁻⁹). Importantly, this signal is not synapse-specific. The 70-gene SynGO synaptic-ribosome set is 98.6% contained within the curated cytoplasmic ribosomal proteome, and the same biphasic inversion runs through GO:CC cytosolic ribosome and the broader translation apparatus — KEGG translation initiation and REACTOME eukaryotic translation elongation both reach TrajDev NES ≈ − 3.0 in both mutations (FDR < 10⁻^13^). The synaptic-localized fraction, therefore, sits at the amplitude extreme of a cytoplasmic structural-ribosome collapse rather than constituting an independent transcriptional program. Chord diagrams and per-gene trajectory heatmaps confirm that dozens of RPL and RPS subunits contribute concordantly to both signals, sharing a temporal pattern of modest upregulation at 35 DIV, strongly negative TrajDev, and pronounced downregulation at 65 DIV across large and small subunits and across both mutations. Per-replicate GSVA analysis establishes that this reversal is a genuine per-sample crossover rather than an artefact of control developmental descent: mutant samples fall from positive group-mean scores at 35 DIV to negative ones at 65 DIV while control samples themselves rise, identifying the structural-pool collapse as an active mutant response. The cytoplasmic ribosome-biogenesis machinery (assembly, processing, nuclear export) runs in the opposite direction at the contrast level — a Compensation signature — but at the per-replicate level this apparent gap-closure is driven by control descent rather than mutant rebound. Biogenesis is therefore attempting compensation at the contrast level while the structural pool decompensates at the per-replicate level. Together, these observations support a model in which DRP1 mutant neurons mount an early compensatory ramp-up of cytoplasmic translation but cannot sustain it through the critical window of synaptogenesis, culminating in a late-stage collapse of cytoplasmic and synaptic translation capacity, a "synaptic ribosome crisis" that is the loudest manifestation of a broader cytoplasmic translation crisis. Additional studies are imperative to understand the connection between mitochondrial dysregulation and this compartmental translation deficit.

We speculate that cortical development and synaptic maturation may be affected in a mutation-specific manner, as evidenced by the notable loss of *PNPO* and *PCDHGC3* expression in G32A cultures but not in R403C cultures. PCDHGC3 isoforms have been found to be required for control of synapse formation, size, and peripheral branching in developing mouse dorsal root ganglia [47]. Additionally, multiple studies have linked gamma protocadherin function to synaptic maturation and stabilization; mutations in these genes, such as protocadherin-19 (*PCDH19*), have to found to increase seizure susceptibility [48–50]. Our findings provide further evidence of gamma protocadherins as possible risk genes for developing childhood encephalopathies. The PNPO enzyme catalyzes the terminal, rate-limiting step in the synthesis of pyridoxal 5’-phosphate, or vitamin B6, a critical cofactor for neurotransmitter synthesis. It is currently unclear how the disruption of the DRP1 GTPase domain activity connects mechanistically to vitamin B6 synthesis. Mutations in *PNPO* have been linked to a similar form of neonatal epileptic encephalopathy, PNPO deficiency [42], in which patients respond to pharmacologic supplementation with pyridoxal 5’-phosphate (PLP) and/or pyridoxine [51–53]. Vitamin B6 supplementation could provide a potentially low-risk therapeutic for patients harboring mutations in specific domains of DRP1. Changes in PNPO and PCDHGC3 at the transcript and protein level provide evidence that defects in specific domains of DRP1 lead to mutation-specific cellular dysfunction that may result in variable patient phenotypes and therapeutic outcomes.

Changes in gene expression of *NNAT*, however, were found in both G32A and R403C cultures compared to controls, suggesting a convergent mechanism of overall DRP1 dysfunction that leads to changes in gene expression. NNAT (neuronatin) was the single most significantly dysregulated gene at 35 DIV in both mutations, with ~ 17–18-fold reductions (G32A logFC = − 4.09, p.adj = 2.7 × 10⁻⁷; R403C logFC = − 4.18, p.adj = 6.4 × 10⁻⁸), and this downregulation persisted to 65 DIV (~ 10–12-fold reduction), a stable transcriptional suppression rather than the recovery dynamic observed for mitochondrial modules. NNAT is a proteolipid that modulates ER calcium homeostasis by antagonizing SERCA, and has been implicated in the regulation of ion channels during brain development [37, 54]. Though its specific role remains unclear, NNAT appears to help regulate intracellular calcium homeostasis. NNAT has been associated with Lafora disease (LD) a teenage-onset inherited progressive myoclonus epilepsy characterized by accumulations of intracellular glycogen inclusions called Lafora bodies [34, 55]. Decreased expression of NNAT in both DRP1 mutants may contribute to a more robust, unconstrained calcium response in neurons, leading to hyperexcitability of synapses and epileptic phenotypes [34, 37].

Synaptic maturation was assessed in mutant DRP1 cultures using super-resolution microscopy of synaptic structures and measurements of functional activity via calcium recording. At 35 DIV, a significant increase in percent overlap between pre- and post-synaptic markers of G32A compared to control synapses indicates a higher density of synapses in these mutant cultures. Ultrastructural studies of synapse development indicate there are mechanisms that ensure, as pre- and post- synapses are modified, they remain proportional to each other [23, 24]. Changes in postsynaptic volumes can subsequently be interpreted as a response to concentrations of neurotransmitter release from the pre-synapse [56, 57]. Decreases in average total volume of G32A 65 DIV synapses (SYP and PSD95 markers) could therefore indicate a possible decline in synaptic activity and overall weakening of these synaptic connections. By 65 DIV, G32A cultures had normalized to a similar synaptic density compared to the control. R403C post-synapses were found to be significantly decreased in total volume at 35 DIV; however, this change was not persistent up to 65 DIV, where post-synaptic total volume was similar to control. Patterns of typical synaptic development have shown that weak synapses are less likely to develop, resulting in a positive selection for stronger synapses [58, 59]. It is likely that cells with weak post-synaptic growth at earlier timepoints are less likely to develop in R403C cultures, with only more proportionately developing synapses remaining by 65 DIV. Although pre-synapses were not significantly different, the culture system and potential need for increased maturation may limit the assessment of synapse integrity in mutant DRP1 neurons. Although pre-synapses were not significantly different, the culture system and potential need for increased maturation may limit the assessment of synapse integrity in mutant DRP1 neurons.

Calcium recordings of 65 DIV mutant DRP1 cultures showed a significantly increased peak amplitude of intracellular calcium transients in response to glutamate stimulation. In addition, both mutant groups showed decreased time to peak response from stimulation, with R403C cultures having a significantly faster response time compared to the control. This increase in rate and magnitude of calcium response may be related to significant downregulation of *CACNG3*, which aids in inhibitory control of AMPARs. It is notable that *CACNG3* is a reported risk gene for a form of childhood epilepsy [60]. Without this inhibition, it is possible that dysregulation of channel opening leads to faster and increased influx of calcium into the cells, resulting in a more robust fluorescence response in DRP1 mutant cells compared to control. Calcium influx into the cell is tightly controlled to maintain intracellular sensitivity to acute fluctuations in calcium concentration necessary for neuronal activity. The mitochondrial network in G32A and R403C mutant DRP1 neurons is fission defective; therefore, much less dynamic than a typical neuronal mitochondrial network. Dynamic mitochondria are required for effective calcium buffering in the cell, so mutant DRP1 neurons are likely less capable of compensating for intracellular calcium dysregulation. This is supported by our findings that mutant DRP1 cells show an increased area under the curve from peak to 60 s (G32A cells having a significantly higher area), which indicates a slower clearance of calcium from the cytoplasm. While calcium efflux is mainly mediated by transporter and channel activity, including the endoplasmic reticulum, mitochondrial calcium buffering is also important in this process. The changes in calcium were accompanied by increased mitochondrial membrane potential in mutant DRP1 cells. This feature is critical because it has recently been exploited for high-throughput screenings in other mitochondrial rare diseases [61].

A growing body of literature has established that mitochondrial metabolism plays a critical role in regulating neuronal differentiation. The transition from neural progenitor to mature neuron is accompanied by a shift from glycolytic to oxidative metabolism, and several groups have demonstrated that mitochondrial dynamics are integral to this process [1, 62, 63] and that mitochondrial morphology directly regulates neural stem cell fate decisions, with mitochondrial fusion promoting differentiation and fission favoring self-renewal. Similarly, previous work has shown that the metabolic state of iPSC-derived progenitors influences not only whether differentiation occurs, but also the tempo at which it proceeds [63, 64]. In this context, the impaired neuronal differentiation observed in DRP1 mutants G32A and R403C is consistent with this established framework. DRP1-mediated fission is required for the mitochondrial remodeling that accompanies the glycolytic-to-oxidative transition during iPSC-to-neuron differentiation. Disruption of DRP1 GTPase activity, as seen with G32A, or altered assembly dynamics as with R403C, compromises the mitochondrial network reorganization necessary to support the increased oxidative phosphorylation demand of differentiating neurons. The impairment in differentiation and maturation observed here, therefore, likely reflects, at least in part, a failure to execute this metabolic switch at the appropriate developmental stage. Future work quantifying oxygen consumption rates and glycolytic flux at defined differentiation timepoints would help clarify the extent to which metabolic reprogramming is disrupted by these mutants, and whether the differentiation phenotype is a direct consequence of metabolic insufficiency or reflects parallel roles of Drp1 in mitochondrial quality control and calcium handling during neurogenesis.

In summary, this study used human iPSC-derived cortical cultures with CRISPR-induced heterozygous patient mutations in either the GTPase or stalk domain of DRP1 to study neuronal maturation and synaptic function. Our main findings show significant perturbations to mitochondrial trafficking, synaptic density, and calcium activity during differentiation and development of these cultures, which occur in a mutation-specific manner. While the exact mechanism linking the mitochondrial defects to the altered transcriptional programs remains unknown, it is plausible that fission disruption triggers metabolic signals that are conveyed to the nuclear transcription machinery for adaptation and survival. Our trajectory analyses point to an additional candidate mechanism: that DRP1's fission defect impairs the delivery of mitochondria into distal neuronal compartments rather than abolishing mitochondrial function per se. This may result in locally energy-depriving the synapse, leaving bulk mitochondrial gene programs transcriptionally intact while precipitating the late-stage collapse of cytoplasmic and synaptic translation. The compartmental asymmetry observed in the RNA-seq data — bulk mitochondrial Compensation alongside cytoplasmic and synaptic structural-ribosome Sign reversal — together with the size-dependent mitochondrial trafficking defects observed by live imaging, are consistent with such a distribution-deficit model. Mechanistically resolving this hypothesis, distinguishing a transport defect from an intrinsic functional defect, will require direct measurement of local ATP availability and local translation at the synapse.

Notable changes in the transcriptional landscape of mutant DRP1 cultures identify risk genes commonly associated with other forms of childhood encephalopathies, highlighting convergent cellular pathways that lead to similar synaptic disorders. Growing understanding of these susceptibility loci increases the likelihood of early detection and access to appropriate therapeutic strategies for affected patients. Identifying DRP1 mutation-specific cellular phenotypes may help contribute to more personalized therapeutics to treat specific symptoms and increase the lifespan of children and adolescents with these and other forms of encephalopathies.

### Limitations

The neuronal cultures used throughout this study were generated from iPSCs, an in vitro system that doesn’t fully recapitulate the complexity of the developing human brain. The in vitro environment likely underestimates certain phenotypes, including effects on presynaptic structure, which may require more mature cultures or additional synaptic markers to be fully resolved. Accordingly, additional studies in more mature neuronal systems, or using complementary approaches such as iNeurons, will be needed to confirm our observations.

While we report significant changes in gene expression and synaptic marker distribution, we do not provide a direct mechanistic link between mitochondrial hyperfusion and altered gene transcription. We speculate that changes in mitochondrial size trigger metabolic signals that influence transcriptional programs; however, this hypothesis remains to be tested empirically and is the subject of ongoing work in our and other laboratories.

The transcriptomic analysis is based on a modest number of biological replicates, with four independent differentiations per genotype per time point. The interaction contrasts, which model mutation-specific changes in maturation trajectory, were underpowered to detect individual differentially expressed genes at stringent thresholds; pathway-level conclusions from gene set enrichment analysis are therefore emphasized over individual gene-level findings.

Finally, although we consistently observe fewer neurons in mutant cultures than in controls, we were unable to determine definitively whether this reflects impaired differentiation of neural progenitor cells, selective neuronal death, or delayed maturation. Cleaved caspase-3 staining did not reveal increased apoptosis, and the deficit appeared to normalize by Day 65, suggesting delayed rather than failed neurogenesis; however, this interpretation requires further investigation.

## Methods

Key Resources Table.

**Table:** 

qPCR Primers	
	5’- > 3’ sequence
MAP2_FW	CTCAGCACCGCTAACAGAGG
MAP2_R	CATTGGCGCTTCGGACAAG
TUBB3_FW	GGCCAAGGGTCACTACACG
TUBB3_R	GCAGTCGCAGTTTTCACACTC
TBR1_FW	GCAGCAGCTACCCACATTCA
TBR1_R	AGGTTGTCAGTGGTCGAGATA
GAPDH_FW	ACAACTTTGGTATCGTGGAAGG
GAPDH_R	GCCATCACGCCACAGTTTC
GPI_FW	GTGTACCTTCTAGTCCCGCC
GPI_R	GGTCAAGCTGAAGTGGTTGAAGC

**Table:** 

Reagent or Resource	Source	Identifier
Antibodies		
Primary Antibodies (Immunocytochemistry)		
Mouse anti-MAP2	Thermo Fisher Scientific	Cat # 131500, AB_2533001
Mouse anti-mitochondria [113–1]	Abcam	Cat#Ab92824 RRID:AB_10562769
Rabbit anti-TUBB3	Abcam	Cat#ab18207 RRID: AB_444319
Rabbit Anti-SYP [YE269]	Abcam	Cat#ab32127 RRID:AB_2286949
Mouse Anti-PSD95	NeuroMabs	Cat#75–028-020 RRID:AB_2877189
Secondary Antibodies (immunocytochemistry)		
Alexa Fluor-488 anti-rabbit IgG	Thermo Fisher Scientific	Cat#A-21206, RRID:AB_2535792
Alexa Fluor-488 anti-mouse IgG	Thermo Fisher Scientific	Cat#A-21202, RRID:AB_141607
Alexa Fluor-568 anti-rabbit IgG	Thermo Fisher Scientific	Cat#A10042, RRID:AB_2534017
Alexa Fluor-568 anti-mouse IgG	Thermo Fisher Scientific	Cat#A10037 RRID:AB_11180865

**Table:** 

Primary Antibodies (Western Blot)		
Rabbit Anti-PNPO	Proteintech	Cat#15552–1-AP RRID:AB_2165814
Mouse Anti-PCDHGC3 [D-11]	Santa Cruz Biotechnology	Cat#sc-376885 AB_3413386
Rabbit Anti-NNAT	Abcam	Cat#ab27266 RRID:AB_776717

**Table:** 

Dyes		
Fluo-4, AM, cell permeant	Invitrogen	Cat#F14201
Pluronic F-127 (20% Solution in DMSO)	Invitrogen	Cat# P3000MP
Mitotracker Green FM	Invitrogen	Cat# M7514
TMRE	Invitrogen	Cat# T669
Cal-520®, AM	AAT Bioquest	Cat# 21130

### Cell lines

The PGP-1 (GM2338) iPSC line used in this study was edited for DRP1 studies. The GM23338 (RRID: CVCL_F182) line is from a 55-year-old healthy, Caucasian male. In addition to one control cell line, two clones were used for each mutation (G32A and R403C). The cells were maintained on Matrigel-coated plates (Corning #354277) and cultured in StemFlex media (Thermo Fisher Scientific #A3349401) at 37 °C with 5% CO2. Culture medium was changed daily. Cells were checked daily for differentiation and were passaged every 3–4 days. For passaging, iPSCs were dissociated with Gentle Cell Dissociation (Stem Cell Technologies #100–0485) and incubated at room temperature for 4 min. All experiments were performed under the supervision of the Vanderbilt Institutional Human Pluripotent Cell Research Oversight (VIHPCRO) Committee. Cells were checked for contamination periodically.

### Differentiation of iPSCs into cortical neurons

Cortical neurons were generated from PGP-1 iPSCs using the dual SMAD neural induction protocol published by Chambers et al. (2009; PMID: 19252484), using 10 mM of SB4 and modified to use 0.4 mM LDN (Stemgent #04–0074) instead of Noggin. The iPSCs were dissociated via incubation in Accutase (Innovative Cell Technologies #AT104) for 8 min at 37 °C and 5% CO_2_. They were then resuspended in StemFlex (Thermo Fisher Scientific #A3349401) media with 10 mM of ROCK inhibitor (Y27632; Stem Cell Technologies #72304) and centrifuged at 200x*g* for 3 min. Cells were plated at 100,000 cells/mL, and neural induction was started when iPSCs reached 100% confluency. For days 0–4 of differentiation, cells are maintained in neuralization media: 410 mL Knockout DMEM/F12 (Invitrogen #12660012), 75 mL Knockout Serum Replacement (Thermo Fisher Scientific #108280238), 5 mL Glutamax (Thermo Fisher Scientific #35050061), 5 mL Penicillin–Streptomycin (Thermo Fisher Scientific #15070063), 5 mL MEM Non-Essential Amino Acids (Thermo Fisher Scientific #11140050), and 1.93 mL of b-mercaptoethanol (BioRad #1610710).

Beginning on day 5, cells are maintained in an increasing concentration of N2 media (Supplementary Fig. 1) 500 mL DMEM/F12 (Thermo Fisher Scientific #11330032), 0.775 g D-Glucose, and 5 mL N2 Supplement (Thermo Fisher Scientific #17502048).

Neural differentiation is continued after 10 days of neural induction (Shi et al.). After day 10, cells are maintained in 50% N2 CTX media and 50% B27 Neurobasal media. N2 CTX media: 500 mL DMEM/F12 + Glutamax (Thermo Fisher Scientific #10565018), 5 mL N2 supplement, 5 mL MEM Non-Essential Amino Acids, and 1.93 mL of b-mercaptoethanol. B27 neurobasal media: 500 mL Neurobasal Media (Thermo Fisher Scientific #21103049), 10 mL B27 supplement (Thermo Fisher Scientific), and 5 mL Glutamax (Thermo Fisher Scientific #17504044).

Cells were replated on days 21 and 35 a differentiation at 500,000 cells/mL for downstream experiments. Cells planned for calcium assays were co-cultured with P1 mouse glia at an ~ 3:1 ratio (neurons:glia).

### Immunofluorescence

Day 35 cells were fixed with 4% paraformaldehyde for 20 min and permeabilized in 1% Triton-X-100 for 10 min at room temperature. After blocking in 10% BSA, cells were treated with primary and secondary antibodies using standard methods. Day 65 cells were fixed in 4% paraformaldehyde for 30 min at 4 C and permeabilized/blocked in 5% Donkey Serum, 0.3% Triton X-100 for 1 h at room temperature. Cells were mounted in Fluoromount-G (Electron Microscopy Sciences #17,984–25) prior to imaging. Primary antibodies used include mouse anti-Mitochondria (Abcam #ab92824), anti-TUBB3 (Abcam #ab18207), anti-SYP (Abcam #ab32127), anti-PSD95 (Neuromabs #75–028-020), anti-MAP2 (Thermo #131,500). Secondary antibodies used include donkey anti-mouse Alexa Fluor-488 (Thermo Fisher Scientific #A21202), donkey anti-rabbit Alexa Fluor-488 (Thermo Fisher Scientific #A21206), and donkey anti-mouse Alexa Fluor-568 (Thermo Fisher Scientific #A10036), and donkey anti-rabbit Alexa Fluor-568 (Thermo Fisher Scientific #A10040). Super-resolution images were acquired using Nikon SIM microscope equipped with a 1.49 NA 100 × Oil objective and Andor DU-897 EMCCD camera. Quantification of microscopy images was performed in NIS-Elements (Nikon) or Fiji. For mitochondrial morphology in neuronal projections, an ROI was manually drawn followed by segmenting mitochondria in 3D and performing skeletonization of the resulting 3D mask. Skeleton volume, surface area, and elongation measurements were exported into Excel.

For video quantifications, time-lapse videos were imported into FIJI ImageJ, and max projections were transformed into single frames using the 3D projection function. Single-frame max projection images were imported into FIJI ImageJ, and an unsharp mask filter was applied. ROIs were created to only include mitochondria from neuronal projections, and mitochondrial network features were analyzed using the Mitochondrial Network Analysis (MiNA) plug-in in FIJI Image J. For quantification of PSD95 and SYP puncta, NIS-Elements GA3 analysis was used to segment binary images in 3D. An intensity threshold was applied, and puncta were filtered by size independently for each marker to calculate total volume. Co-localization of PSD95 and SYP was determined using the Boolean AND function to calculate volumetric overlap between the two channels. To establish a random co-localization baseline, a custom Python script (https://github.com/CostanzoJa/Neuronal-Analyses.git) was developed in which the PSD95 channel was systematically rotated by 90° and 180°. Co-localization at each rotation was then quantified via binary segmentation, mask creation, and 3D volumetric analysis across all Z-planes to determine the extent of overlapping puncta by chance alone. Representative images were prepared using Affinity Designer 2 (affinity.serif.com/en-us/designer/)*.*

### Immunoblots

Cells were harvested in lysis buffer: 1% Triton, 10X phosphatase inhibitor (PhosSTOP; Roche, Sigma Aldrich #4,906,837,001), 10 × protease inhibitor (PIC; Roche, Sigma Aldrich #04693159001), and 100X phenylmethylsulfonyl fluoride (PMSF) (RPI #P20270). Lysate protein concentrations were quantified using BCA analysis (Fisher Scientific #23225). Western blotting was performed using the Bio-Rad Mini-PROTEAN Electrophoresis System (Bio-Rad #1658036). Samples were run on either 4–20% or 12% Mini-PROTEAN TGX gels (Bio-Rad #4,561,094/#4,561,044). Protein was transferred from gels onto methanol-activated PVDF membranes (Bio-Rad #1620177). All membranes were washed in Tris-buffered saline with 0.1% Tween-20 (TBST) and blocked in TBST containing 5% milk. Primary antibodies were incubated at 1:1,000 overnight at 4 °C unless otherwise stated. The following secondary antibodies were used at 1:10,000 dilution and incubated for 1 h at room temperature in 5% milk w/TBST: HRP donkey anti-rabbit (Jackson ImmunoResearch #711–035–152) and HRP goat anti-mouse IgG subclass 1-specific (Jackson ImmunoResearch #115–035–205). Protein signal was developed using either Pierce ECL Western Blot Substrate (Thermo Fisher Scientific #32,109) or SuperSignal West Femto (Thermo Fisher Scientific #34,094), depending on the sensitivity of the primary antibody. Protein signal was visualized using the Amersham Imager 600 (GE Life Sciences #29–0834-61). Images were quantified using Fiji (imagej.net/software/fiji/), to measure the integrated density of each band, subtracting the background and normalizing to the loading control. Representative images were prepared using Affinity Designer 2 (affinity.serif.com/en-us/designer/)*.*

### RNA extraction and cDNA synthesis

Cells were washed with PBS and scraped into 1000 μl Trizol reagent. 200 μl of chloroform was added, and the samples were incubated at room temperature for 5 min. The samples were centrifuged at 12,000 g, and the aqueous phase was collected. 500 μl of isopropanol was added to precipitate RNA, and the sample was incubated for 25 min at room temperature, followed by centrifugation at 12,000 g for 10 min at 4 °C. The RNA pellet was washed with 75% ethanol, semi-dried, and resuspended in 30 μl of DEPC-treated water. After quantification and adjustment of the volume of all the samples to 1 μg/μl, the samples were treated with DNAse (New England Biolabs #M0303). 10 μl of this volume was used to generate cDNA using the manufacturer’s protocol (Thermo Fisher #4368814).

### Quantitative RT PCR (RT-qPCR)

1ug of cDNA sample was used to run RT-qPCR for the primers mentioned in the key resources table. QuantStudio 3 Real-Time PCR machine, SYBR green master mix (Thermo Fisher #4364346), and manufacturer instructions were used to set up the assay. Housekeeping genes *GAPDH and GPI* were used for normalization.

### Detailed transcriptomic analyses

Twenty-five biological replicates spanning six experimental groups (3 genotypes × 2 timepoints; n = 3–6 per group) were analyzed. All downstream analyses were conducted on the filtered, quality-controlled dataset. The details are provided below.

### Reads processing, QC and alignment

Reads were trimmed with Trimmomatic [65] v0.39 to remove adapters and low-quality bases. Paired-end FASTQ files were aligned to the human GRCh38.p14 RefSeq assembly (GCF_000001405.40; annotation from the same release). We used STAR [66] to build the index and align the reads in two-pass mode, followed by coordinate sorting. Library quality was assessed pre- and post-alignment with RSeQC [67] (infer_experiment.py; libraries determined non-stranded), Picard [68] (CollectRnaSeqMetrics, MarkDuplicates), samtools [69] (flagstat) and MultiQC [70] for aggregation. Gene-level quantification used featureCounts [71] (Subread), summarizing exons to gene_id in paired-end, non-stranded mode.

All shell invocations (STAR index/alignment, post-alignment QC, featureCounts) were executed inside a Docker image built from the public recipe scbio-docker (.devcontainer/Dockerfile.dev), tagged scdock-r-dev:v0.2 (R 4.4.2, Python 3.10, and common NGS tools including STAR 2.7.11b, featureCounts 2.0.2, samtools/bcftools/htslib 1.21, FastQC 0.12.1, Trimmomatic 0.39, Picard). The exact build arguments and package set are documented in the container recipe.

### Downstream analysis

Downstream analyses were performed in R [72] 4.5.0 and Python 3.10.12 We performed differential expression analysis in the edgeR [73, 74] and limma-voom [75, 76] framework.

Lowly expressed genes were filtered with edgeR::filterByExpr() (with default parameters: min.count = 10, min.total.count = 15, large.n = 10, min.prop = 0.7, implementing a design-aware heuristic that requires ≥ 10 counts in ≥ 70% of samples within the smallest group — i.e. ≥ 3 biological replicates per genotype × time combination in the present design). Library sizes of the 21,122 retained genes were then normalized by Trimmed Mean of M-values (TMM).

### Statistical modelling

Our primary goal was to describe how the genotype effect evolves over development. We fit a factorial design matrix encoding genotype × timepoint combinations with voomLmFit() with no sample weights moderation (sample.weights = FALSE). We applied the robust empirical Bayes method to prevent outlier gene variances from biasing the global variance trend, particularly given the unbalanced design.

We defined nine contrasts to address three biological questions.

Mutation effects at each developmental stage

Mutant vs control within a single timepoint:

- ◦ early (D35): G32A_vs_Ctrl_D35, R403C_vs_Ctrl_D35.
- ◦ late (D65): G32A_vs_Ctrl_D65, R403C_vs_Ctrl_D65

Positive logFC indicates higher expression in mutant versus control at the defined timepoint.

- Maturation within each genotype (D65 − D35)

Track developmental changes, pair-wise comparisons between timepoints within a genotype:

- Time_Ctrl = Ctrl_D65 – Ctrl_D35,
- Time_G32A = G32A_D65 – G32A_D35,
- Time_R403C = R403C_D65 – R403C_D35

These contrasts describe normal developmental maturation (*Time_Ctrl*) and how maturation proceeds in each mutant (*Time_G32A, Time_R403C*). Positive logFC indicates higher expression at D65 versus D35 within the given genotype. These contrasts were used to confirm expected developmental patterns in controls and to aid interpretation of interaction effects but are not explicitly plotted in all of the main figures (see Supplements for full DE tables for all contrasts tested).

## Mutation-specific trajectory deviations (TrajDev, interaction term)

To test whether mutant maturation deviates from normal development, we used defined difference-in-difference interaction:

- Maturation_G32A_specific = Time_G32A − Time_Ctrl.
- Maturation_R403C_specific = Time_R403C − Time_Ctrl.

These interaction contrasts capture whether the mutation alters the normal maturation trajectory.

Results and figures are organized as *Early* (D35 mutation effects), *TrajDev* (mutation-specific trajectory deviation from normal maturation), and *Late* (D65 mutation effects) to characterize developmental dynamics.

Positive logFC in these contrasts indicates genes or pathways where the mutation causes a greater increase or smaller decrease over time compared to controls; negative logFC indicates a smaller increase or greater decrease than normal maturation. Importantly, there is no algebraic requirement that TrajDev oppose normal maturation. Apparent "inverse" patterns (e.g., positive Time_Ctrl, negative TrajDev) arise only when controls change strongly over time and mutants change little or in the opposite direction. The sign and magnitude of TrajDev therefore captures genuine biological differences between mutant and control trajectories. A significant (FDR < 0.05, |NES|> 0.5) TrajDev term indicates non-parallel maturation trajectories between mutant and control and identifies pathways where the mutant diverges from the control trajectory.

For each contrast, we applied Benjamini–Hochberg FDR correction. Unless otherwise noted, per-gene significance was assessed at FDR < 0.05 (BH-adjusted); pathways were considered enriched at the same threshold. A secondary exploratory analysis of all nine contrasts at FDR < 0.10 is reported where noted. Complete limma tables of differentially expressed genes for every contrast (all genes) and the master pathway table (including all contrasts) are included in Supplementary Tables (Supplementary Table S1_DE).

For each contrast we applied Benjamini–Hochberg (BH) FDR correction. A secondary exploratory analysis of all nine contrasts at FDR < 0.10 is reported where noted (script 02_Analysis/generate_DE_counts_FDR_0.1.R) and is used in Supplementary Fig. S6B as a threshold-robustness check for the maturation-contrast overlap. Complete limma tables of differentially expressed genes for every contrast (all genes) and the master pathway table (including all contrasts) are included in Supplementary Tables (Supplementary Table S1_DE).

### Gene set enrichment analysis

Pathway inference used GSEA to avoid dependence on any hard per-gene cutoff. For each contrast, all genes that passed filterByExpr() were ranked by the moderated t-statistic, and clusterProfiler [77–79] (fgsea [80] backend) was used with 100,000 permutations per contrast and FDR control. We queried MSigDB [81] (Hallmark, Gene Ontologies (GO) Biological Processes (BP) [82], GO Cellular Components (CC), GO Molecular Functions (MF), Reactome [83], KEGG [84], WikiPathways [85], Chemical and Genetic Perturbations, Transcription Factor Targets) via msigdbr [86]. To provide focused coverage of the two organelle compartments most relevant to DRP1 biology, we additionally queried SynGO [22] (synaptic gene ontologies) and MitoCarta 3.0 [87] (mitochondrial pathways), applying identical gene-set size filtering (10–500 genes) and the same fgsea pipeline. Together these 12 collections yielded 12,221 gene sets. Of these, 5,267 reached FDR < 0.05 in at least one of the nine GSEA contrasts (Early, TrajDev, or Late for either mutation, plus the three time-course contrasts); this 'ever-significant' set is the primary analysis universe for pattern classification and all downstream distributional summaries. Figures report NES and BH-adjusted FDR; dot plots encode − log10(FDR) by bubble size and NES direction by color.

### Trajectory pattern classification

To summarize the shape of pathway enrichment trajectories across the three contrast terms, each pathway was assigned to one of eight mutually exclusive, hierarchically ordered pattern labels based on the NES and FDR values of its Early, TrajDev, and Late components (defined in Statistical modelling section above):

- Early: mutant vs control at D35
- Trajectory deviation (TrajDev): mutation-specific deviation from normal maturation
- Late: mutant vs control at D65

Pathways were classified into discrete patterns based on the statistical significance (FDR < 0.05, |NES|> 0.5) and direction of the Early, TrajDev, and Late components. Patterns are mutually exclusive and assigned in a hierarchical order. They fall into three groups.

- A. Active Patterns (require significant TrajDev)
  These patterns require:
  - Significant Early defect (FDR < 0.05, |NES_Early|> 0.5)
  - Significant TrajDev (FDR < 0.05, |NES_TrajDev|> 0.5)
- A1. Compensation
  - TrajDev opposes Early (opposite sign)
  - Late improved (≥ 30% reduction in |NES| or |Late|< 0.5)
  - Interpretation: active adaptive plasticity partially or fully counteracts the initial defect.
- A2. Sign_reversal
  - TrajDev opposes Early
  - Late flips sign relative to Early with |NES_Late| > 0.5
  - Interpretation: complete trajectory reversal.
  - Biologically this can represent:
    - ◦ failed compensation (e.g. Early up → Late down in synaptic ribosomes),
    - ◦ or eventual recovery (e.g. Early down → Late up in mitochondrial pathways), depending on context.
- A3. Progressive
  - TrajDev amplifies Early (same sign)
  - Late worsened (|Late|/|Early|> 1.3)
  - Interpretation: active maladaptive response where development drives further away from normal.
    Importantly, Progressive category was not observed in either mutation, and so is not mentioned in the Results section.
- B. Passive Patterns (no significant TrajDev)
  Here, Early shows a significant defect, but TrajDev does not reach significance (FDR ≥ 0.05 or |NES_TrajDev|≤ 0.5):
- B1. Natural_improvement
  - Late improved (≥30% reduction or |Late| < 0.5)
  - Interpretation: the defect improves over time, consistent with normal developmental buffering rather than mutation-specific programs.
- B2. Natural_worsening
  - Late worsened (|Late|/|Early| > 1.3)
  - Interpretation: the defect passively worsens during maturation without clear evidence of active adaptation.
- C. Other Developmental Patterns
- C1. Late_onset
  - No Early defect (FDR ≥ 0.10 or |NES_Early|≤ 0.5)
  - Strong Late defect (FDR < 0.05, |NES_Late|> 1.0)
  - Interpretation: maturation-dependent dysfunction; pathways appear normal at D35 but become abnormal at D65.
- C2. Transient
  - Strong Early defect (FDR < 0.05, |NES_Early|> 1.0)
  - Late resolved (|NES_Late|< 0.5)
  - Interpretation: early disruption that fully recovers by D65 (developmental delay).
- C3. Complex
  - Trajectories that do not meet any of the above criteria, e.g. multiphasic, weak, or inconsistent patterns.

While Time_Ctrl (control maturation) is implicit in TrajDev's calculation, our pattern classification focuses on the net deviation (TrajDev) as the primary metric for detecting trajectory abnormalities, i.e. the sign of TrajDev is interpreted relative to the Early defect. For pathways where biological interpretation requires understanding the normal maturation program (Time_Ctrl), these values are provided in the supplementary data (Supplementary Table S2, columns: NES_Time_Ctrl, p.adjust_Time_Ctrl).

This hierarchical decision tree partitions a three-dimensional NES space (NES_Early × NES_TrajDev × NES_Late) into biologically named regions. In the two-dimensional projection onto the (NES_Late, NES_Early) plane patterns that share the same quadrant are distinguished by the TrajDev significance gate (color), while Sign_reversal and Transient/Late_onset are separated by whether the Late NES changes quadrant or approaches zero (Supplementary Fig. S12).

The pattern boundaries correspond to biologically motivated criteria: a 30% reduction in effect magnitude between Early and Late stages (improvement ratio < 0.7) as a conventional threshold for meaningful change, a sign flip between Early and Late for Sign_reversal, and |NES|> 0.5 as a standard minimum for claiming a biologically relevant enrichment signal. These criteria partition the (NES_Late, NES_Early) plane into interpretable pattern regions, conditioned on TrajDev significance, and are visualized geometrically in Supplementary Fig. S12. The classifier is descriptive—it summarizes the three-dimensional NES space (defined by the NES_Late, NES_Early in 2 dimensions, and the TrajDev_NES as the third dimension) using conservative, pre-specified boundaries and does not introduce new inferential tests beyond the per-stage GSEA enrichment p-values. Compensation, Sign_reversal, and Progressive each require a statistically significant TrajDev (FDR < 0.05, |NES_TrajDev|> 0.5) and are therefore strict subsets of the interaction-contrast-significant pathways; the classifier partitions those pathways into directionally interpretable categories without adding new pathways to the universe. Late_onset is a distinct category defined by the absence of an Early defect and the presence of a strong Late defect (|NES_Late|> 1.0, FDR < 0.05); TrajDev significance is neither required nor excluded. Robustness to threshold choice was assessed by re-running the pattern assignment across a grid of 81 threshold combinations (NES_EFFECT ∈ {0.4, 0.5, 0.6}; NES_STRONG ∈ {0.8, 1.0, 1.2}; IMPROVEMENT_RATIO ∈ {0.6, 0.7, 0.8}; WORSENING_RATIO ∈ {1.25, 1.3, 1.4}; restricted to the same 5,267 ever-significantly-enriched pathways (i.e., FDR < 0.05 in at least one of the nine GSEA contrasts—the denominator used throughout the Results section). This universe is broader than the active-responder subset and was used precisely because it holds constant while the TrajDev significance threshold varies within the grid. Across all 81 combinations, the principal claims hold: Compensation exceeds passive-recovery patterns (Natural_improvement + Natural_worsening) in both mutations; Progressive patterns are absent in both; R403C consistently shows more Compensation than G32A; and Compensation is the single largest active pattern in both mutations **(**Supplementary Table S4; Supplementary Table S9; Supplementary Table S10). The pattern taxonomy does not constitute new formal hypothesis testing; it is a structured summary of the NES and FDR values already generated by the GSEA contrasts.

All 12,221 pathways tested in GSEA receive a pattern classification. For distributional summaries we report counts within the 5,267-pathway ever-significant universe (FDR < 0.05 in at least one contrast; designated 'ever_significant' in Supplementary Table S2). For focused analysis of pathways with statistically confirmed trajectory deviation, we additionally define an active-responder subset: pathways within the ever-significant universe that carry a significant TrajDev (FDR < 0.05, |NES_TrajDev|> 0.5), yielding 2,039 pathways in G32A and 2,242 in R403C. For trajectory-curve visualization (bump chart, Fig. 5B), we include 4,142 pathways that received a non-Complex pattern in at least one mutation; this set encompasses the 3,751 ever-significant pathways with a non-Complex label plus 391 pathways that narrowly miss the ever-significant threshold (0.05 ≤ FDR < 0.10 in at least one contrast) but nonetheless attain a non-Complex pattern label — these are retained to avoid discarding pathways with biologically coherent trajectory shapes.

### Per-replicate pathway enrichment (GSVA)

To name the mechanism underlying each GSEA pattern at the replicate level — specifically, to distinguish whether a trajectory change in the contrast reflects mutant and/or control trajectory dynamics — we computed Gene-Set Variation Analysis (R library GSVA 2.2.0 [88]) scores in parallel to the contrast-based GSEA. GSVA was run with the GSVA Bioconductor package on the same logCPM matrix used for differential expression (gsvaParam with kcdf = "Gaussian", minSize = 10, maxSize = 500; identical gene-set size filter to GSEA), yielding a per-sample enrichment score for each of 14,500 gene sets drawn from the same.gmt inputs as the GSEA analysis (Hallmark, GO:BP/CC/MF, Reactome, KEGG, WikiPathways, CGP, TF targets, MitoCarta 3.0, SynGO). Group-level inference used Welch's t-tests on per-sample GSVA scores comparing each mutant to control within each timepoint, with Benjamini–Hochberg FDR correction. Pathways were assigned a parallel GSVA pattern label using the same hierarchical algorithm as in (e), with magnitude thresholds rescaled to the GSVA score range: GSVA_EFFECT = 0.15 (analogous to |NES|> 0.5) and GSVA_STRONG = 0.30 (analogous to |NES|> 1.0). Significance and improvement/worsening-ratio thresholds were unchanged. The rescaled magnitudes preserve the " ≥ 30% effect-size change" semantics of the GSEA classifier while accounting for GSVA's bounded score range (|score|≤ 1) versus NES (unbounded). The GSEA and GSVA pattern classifications are reported in parallel rather than merged: GSEA carries direct multiple-testing-corrected significance for the interaction (TrajDev) contrast, while GSVA carries replicate-level visualisation of the enrichment trajectory underneath each label. Where the two classifications disagree for a given pathway, the disagreement itself is the headline—the per-replicate panel (click-through panel in Supplementary Data File 1 for every classifiable pathway) lets the reader inspect the sample-level data directly.

To provide a categorical summary of the arm-level dynamics visible in the GSVA per-replicate view, we implemented a GSVA driver classification. For each pathway × mutation combination, we computed the per-arm delta: Δ_arm = median GSVA score at D65—median GSVA score at D35, separately for Ctrl and for the mutant genotype. Arms with |Δ|≥ ε = 0.10 were considered 'moving' (ε chosen as a conservative lower bound on a biologically detectable shift in the GSVA score range [0, 1]). Pathways were then assigned one of four labels: `mutant_driven` (only mutant arm moves), `ctrl_driven` (only Ctrl arm moves), `both_moving` (both arms move), or `neither_moving` (neither arm clears the threshold). Pathways outside the GSVA gene-set size filter (minSize = 10, maxSize = 500) receive a null label. These labels are embedded in the interactive dashboard as a sidebar filter and as a verdict block in each pathway's click-through modal, allowing the reader to isolate, for example, all `ctrl_driven` Compensation pathways—i.e., pathways where the GSEA Compensation pattern is driven by Ctrl developmental descent rather than mutant rebound. The driver classification is descriptive and uses a single threshold (ε = 0.10); it does not constitute a formal statistical test.

### Targeted calcium-signaling gene analysis

A curated set (NNAT, CACNG3, CACNA1C, CACNA1S, ATP2A1, RYR1, MYLK3, CASR, VDR, STIM1, STIM2, ORAI1, CALB1, CALR, PNPO) was tracked. Genes not detected above `filterByExpr` expression thresholds (CACNA1C, CASR) were excluded from the figures. In addition, three further members of the set (CACNG3, CACNA1S, VDR) were retained by `filterByExpr` but sit below AveExpr = 0 log2-CPM. These three genes are present in the statistical analysis, in the heatmaps of Fig. 6C (which plot the thirteen-gene set including CACNG3, CACNA1S and VDR, but are not rendered on the AveExpr > 0 volcano panels (Fig. 4A). We report row-scaled heatmaps of log2-CPM and per-gene boxplots by genotype × time (Fig. 6C and Supp. Figure 2D, respectively); these genes are also highlighted in the main volcano panels (Fig. 4A and Supp. Figure 2D).

### Reproducibility, compute environment and availability

Analyses fully scripted and version-controlled. All steps were executed inside a Docker image built from tony-zhelonkin/scbio-docker (tag scdock-r-dev: v0.5.1), which provisions R 4.5.0, Bioconductor 3.21, Python 3.10, and the NGS toolchain (e.g., STAR 2.7.11b, Subread/featureCounts 2.0.2, samtools/bcftools/htslib 1.21, FastQC 0.12.1, Trimmomatic 0.39, Picard). Plotting and GSEA wrappers are adapted from tony-zhelonkin/RNAseq-toolkit.

All code and instructions to rerun the analysis are available at the GitHub repo https://github.com/Mogilenko-Lab/Gama_Vivian_DRP1_bulkRNAseq/tree/v2.1.0 and archived at Zenodo (https://doi.org/10.5281/zenodo.20213748). An interactive self-contained visualization HTML dashboard for exploring pathway trajectories, pattern classifications, and GSEA/GSVA statistics is available at https://drp1.omicable.com/

### Mitochondrial live cell imaging

Day 65 neuronal cultures were incubated in MitoTracker Green FM (Thermo #) at 100 µM for 30 min at room temperature. Cells were washed once with PBS and fresh media was added and imaged immediately. Live recordings of mitochondria in neuronal projections were acquired on Nikon W1 Spinning Disk Confocal 100X plan Apo 1.49 NA. Oil objective and a Prime 95B CMOS camera for ten minutes, acquiring a z-stack every 10 s. All images were acquired using perfect focus, which helps account for axial focus fluctuations in real-time. Live recordings were processed with Richardson-Lucy deconvolution (20 iterations) and denoised using Nikon Elements software. Manual tracking of mitochondria was completed using FIJI manual tracking plugin with centering correction and x/y calibration 0.11 at 100X. All mitochondria within ten cell processes were tracked for each condition. Velocity and distance were measured via the Manual Tracking plugin in Fiji Image J. The plugin provides XY coordinates and velocity, distance covered between two frames and intensity of the selected pixel or volume by clicking on the structure of interest in consecutive frames including a local maximum centering correction. Average distance is the distance covered within one frame, averaged across all frames of the recording, and total distance is the distance covered in the total ten-minute recording.

### Calcium imaging

Fluo-4AM loading protocol adapted from Thermofisher’s protocol for fluo-4-based measurements of cytosolic calcium changes in neural stem cells in response to neurotransmitter application. Day 65 neuronal cultures co-cultured with P1 mouse glia were incubated with 2.86 µM Fluo-4-AM cell permeant dye (Invitrogen F14201) with Pluronic F-127 solution (Invitrogen P10020) in aCSF for 1 h at 37 °C. Cells were washed once with media followed by fresh aCSF media and imaged immediately. Cells were imaged on the Nikon W1 Spinning Disk Confocal 100X Plan Apo 1.49 NA Oil objective and a Prime 95B CMOS camera for 1 min with 150 µM Glutamate (Glu) (Thermo A15031) with stimulation occurring at 15 s. Four wells were imaged per line in each replicate. Live recordings were processed with Richardson-Lucy deconvolution (20 iterations) and denoised using Nikon Elements software. Approximately 50 regions of interest (ROIs) were chosen across all recordings per group; Elements “Tracking” function was used to track mean fluorescence over time across all binaries. Change in fluorescence values were calculated as a function of change in fluorescence over baseline fluorescence (Df/f) plotted over time. For statistical analysis, each G32A and R403C trace was normalized to a matched control for each replicate.

### Basal calcium imaging

Cal-520 AM loading protocol adapted from AAT Bioquest’s protocol for Cal-520 AM-based measurements of cytosolic calcium changes. Day 200 + neuronal cultures were incubated with 5 µM Cal-520 AM cell-permeant dye (AAT Bioquest 21130) for 1 h at 37 °C. Cells were washed once with FluorBrite DMEM (Invitrogen A1896701) followed by fresh FluorBrite DMEM with Hoescht 33,342 (Invitrogen), incubated for 15 min at 37 °C on the confocal scope, and imaged immediately after. Cells were imaged on the Nikon W1 Spinning Disk Confocal 100X Plan Apo 1.49 NA Oil objective and a Prime 95B CMOS camera for 5 min every 10 s. Three wells were imaged per line in each replicate. Live recordings were processed with Richardson-Lucy deconvolution (10 iterations) and denoised using Nikon Elements software. A Python script (https://github.com/CostanzoJa/Neuronal-Analyses.git) was developed to generate masks over cells fluorescing, measuring intensity over time across all binaries. Change in fluorescence values was calculated as a function of change in fluorescence over baseline fluorescence (dF/F0) of the 8th percentile for each recorded video. Relative Cal-520 AM mean fluorescence intensity was calculated through background correction and normalization to the average of controls at time 0, which was graphed over time, and as the mean intensity per replicate. The mean deviation of Cal-520 AM fluorescence intensity was calculated through background correction, change in fluorescence per frame, and normalization to the average of controls at time 0, which was graphed over time, and as the mean intensity per replicate. Representative images were prepared using Affinity Designer 2 (affinity.serif.com/en-us/designer/)*.*

### TMRE imaging

Day 60 neuronal cultures were incubated with 500 nM of TMRE cell-permeant dye (Invitrogen T669) for 1 h at 37 °C. Cells were washed once with FluorBrite DMEM (Invitrogen A1896701) followed by fresh FluorBrite DMEM with Hoescht 33342 (Invitrogen), incubated for 15 min at 37 °C on the confocal scope, and imaged immediately after. Cells were imaged on the Nikon W1 Spinning Disk Confocal 100X Plan Apo 1.49 NA Oil objective and a Prime 95B CMOS camera for 10 min every 10 s. Three wells were imaged per line in each replicate. Live recordings were processed with Richardson-Lucy deconvolution (10 iterations) and denoised using Nikon Elements software. A Nikon Elements GA3 script was developed to measure the fluorescence of TMRE via binary masks, measuring the intensity over time across all binaries through the 3D plane. Relative TMRE mean fluorescence intensity was calculated by background correction and normalization to the average of controls at time 0, and the results were graphed over time and as the mean intensity per replicate. The mean deviation of TMRE fluorescence intensity was calculated by background-correcting the change in fluorescence per frame and normalizing to the average of controls at time 0, which was graphed over time and as the mean intensity per replicate. Representative images were prepared using Affinity Designer 2 (affinity.serif.com/en-us/designer/)*.*

### Sample preparation and image acquisition for transmission electron microscope (TEM) analysis

Neurons were immediately washed in ice-cold saline. Cells were fixed in 2.5% glutaraldehyde in sodium cacodylate buffer for 1 h at 37 °C, embedded in 2% agarose, postfixed in buffered 1% osmium tetroxide, stained in 2% uranyl acetate, and dehydrated with an ethanol graded series. After embedding in EMbed-812 resin, 80 nm sections were cut on an ultramicrotome and stained with 2% uranyl acetate and lead citrate. Images were acquired on a JEOL JEM-1230 transmission electron microscope operating at 120 kV. Random images of neurons were obtained. To assess mitochondrial area and aspect ratio, stitched images were imported into FIJI Image J and ROIs for each mitochondrion not contained within the soma were created and analyzed using the measure area and fit ellipse functions.

### Mitochondrial event localizer (MEL)

The MEL algorithm processes a fluorescence microscopy timelapse sequence of z-stack images of MitoTracker-labeled mitochondria and produces event counts per frame and 3D locations indicating where mitochondrial events are likely to have occurred at each time step [20]. These locations can subsequently be superimposed on the z-stacks to indicate the different mitochondrial events. The algorithm was organized into two consecutive steps, namely the image pre-processing step, which normalizes and prepares the time-lapse frames, and the automatic image analysis step, which calculates the location of the mitochondrial events based on the normalized frames. MEL also allows each event to be subsequently validated and removed from the visualization and event counts. The code is available for download: https://github.com/rensutheart/MEL-Fiji-Plugin.

### Statistical analysis

All experiments were performed with a minimum of 3 biological replicates. Statistical significance was determined by unpaired t-tests with Welch’s correction, paired t-test, and one-way or two-way analysis of variance (ANOVA) with comparisons to control as appropriate for each experiment, unless otherwise noted. Significance was assessed using Dunnett’s post-hoc multiple comparisons test. GraphPad PRISM v9 or higher and Statplus software package were used GraphPad Prism was used for all statistical analysis (SAS Institute: Cary, NC, USA) and data visualization. Error bars in all bar graphs represent standard error of the mean unless otherwise noted in the figure. No outliers were removed from the analyses. For all statistical analyses, a significant difference was accepted when P < 0.05.

## Supplementary Information

Supplementary Material 1.

Supplementary Material 2.

Supplementary Material 3.

Supplementary Material 4.

Supplementary Material 5.

Supplementary Material 6.

Supplementary Material 7.

Supplementary Material 8.

Supplementary Material 9.

Supplementary Material 10.

Supplementary Material 11.

Supplementary Material 12.

Supplementary Material 13.

Supplementary Material 14.

Supplementary Material 15.

Supplementary Material 16.

Supplementary Material 17.

Supplementary Material 18.

Supplementary Material 19.

Supplementary Material 20.

Supplementary Material 21.

Supplementary Material 22.

Supplementary Material 23.

Supplementary Material 24.

## Data Availability

Data is provided within the manuscript or supplementary information files.
