# Supplementary material for: DRP1 mutations associated with EMPF1 encephalopathy perturb the transcriptional profile and maturation of cortical neurons
Source: J Neurodev Disord. 2026 Jun 26;18:52. doi: 10.1186/s11689-026-09713-0 (PMC13563865; doi:10.1186/s11689-026-09713-0)
Supplement: Supplementary file 4 [file 11689_2026_9713_MOESM13_ESM.pdf]

Supplementary Figure Legends

Supplementary Figure S1 | Cortical differentiation timeline and mitochondrial event localizer. (A) iPSCs with DRP1 mutations differentiated in Knock Out Serum Replacement media from day 0 and gradually transitioned into N2 media over 11 days with addition of dual SMAD inhibitory small molecules SB-431521 and LDN-193189. After day 11, cells are transitioned into neural maintenance media for the duration of culture. (B) Cells were imaged using Mitotracker at 5-second intervals for 15 minutes. The data represent the mean across timepoints of the per-timepoint event count divided by the number of segmented mitochondrial structures

Supplementary Figure S2 | Quality control analysis of bulk RNA-seq data. (A) Sample–sample Pearson correlation heatmap across all 25 libraries. All within-group r > 0.85. Anchors the Quality-Control claim in `RESULTS_combio0` Color scale represents correlation coefficient (yellow = high correlation, brown/orange = lower correlation). **(B) Principal component analysis (PCA)** of $\log_2$-CPM values. PC1 (35.1% of variance) separates samples mostly by developmental timepoint (D35 vs D65), while PC2 (15.3% of variance) seems to capture genotype-associated variation within each timepoint. Points are colored by genotype (blue = Control, orange = G32A, green = R403C) and shaped by timepoint (circle = D35, triangle = D65). PCA was performed on scaled data. **(C) Volcano plots for within-genotype maturation contrasts (D65 vs D35)** showing differential gene expression. Each plot displays $\log_2$ fold change (x-axis) versus $-\log_{10}$ adjusted p-value (y-axis). Genes passing FDR < 0.1 are colored (red = upregulated, blue = downregulated); calcium signaling genes (NNAT, CACNG3, CACNA1S, ATP2A1, RYR1, MYLK3, VDR, STIM1, STIM2, ORAI1, CALB1, CALR, PNPO) are highlighted. Maturation induced no significant transcriptional changes in control neurons (Time_Ctrl: 0 DEGs at FDR < 0.05 and FDR < 0.1), but drove distinct expression programs in both DRP1 mutants (Time_G32A: 68 DEGs; Time_R403C: 124 DEGs at FDR < 0.05), suggesting mutation-specific developmental trajectories. **(D) Expression boxplot profiles** Per-gene $\log_2$-CPM boxplots for the 13-gene

curated calcium panel across genotype × timepoint. Each subpanel: one gene; x-axis = group; y-axis = $\log_2$-CPM. Disambiguates whether each panel member behaves consistently across replicates. Each panel shows $\log_2$-CPM values for one gene, with boxplots representing expression distributions across six genotype-timepoint combinations (Ctrl D35, G32A D35, R403C D35, Ctrl D65, G32A D65, R403C D65). Individual biological replicates (n=4 per condition) are overlaid as points. Colors indicate genotype (teal = Control, orange = G32A, purple = R403C).

Supplementary Figure S3 | Volcano plots. AveExpr ≥ 0 volcano panel — counterpart to Fig. 4A expression-floor filter applied. Encoding matches Fig. 4A; filtering to AveExpr ≥ 0 removes low-expression artefacts and shows that the DEG core (NNAT, PNPO, PCDHGC3, etc.) is preserved while CACNA1C, CASR, and several other low-expression panel members drop out.

Supplementary Figure S4 | Comprehensive GSEA Landscape Across All Experimental Contrasts reveals extensive transcriptional remodeling in DRP1 mutant neurons across developmental stages. Dotplot visualization of GSEA results pooled from across all tested pathway databases (n = 10), showing top 5 significantly enriched pathways per database (FDR < 0.05) for each experimental contrast. Layout: **Top row**, Early mutation effects (D35 vs control) for G32A (left) and R403C (middle); **Middle row**, Late mutation effects (D65 vs control) for G32A (left) and R403C (middle); **Bottom row**, Maturation effects (D65 vs D35 within genotype) for G32A (left), R403C (middle), and Control (right). Each dot represents a significantly enriched pathway; horizontal position (x-axis) indicates gene ratio (proportion of leading edge genes in pathway); dot color indicates normalized enrichment score (NES, orange = positive enrichment/upregulation, blue = negative enrichment/downregulation); dot size reflects significance ($-\log_{10}$ FDR, larger dots = more significant); black outline denotes FDR < 0.05. Complete GSEA results for all contrasts and databases are provided in Supplementary Table 2.

Supplementary Figure S5 | Per-database pattern heatmap. Pathway-count heatmap of pattern assignments across the 12 databases × 8 trajectory patterns. Two side-by-side panels for G32A and R403C; cells show the absolute pathway count receiving each pattern label per database. **Companion table: ` per_database_pattern_summary.csv`.**

Supplementary Figure S6 | Focused-panel pattern classification (MitoCarta + SynGO). Pattern-distribution bar chart restricted to the focused 104-pathway panel (72 MitoCarta + 32 SynGO). Of the 104, 39 carry a non-Complex pattern in at least one mutation; the remaining 65 are Complex in both and appear only in the focused view (Fig. 5C).

Supplementary Figure S7 | Replicate-level GSVA. Per-replicate GSVA scores for six representative modules (Synaptic Ribosome, Cytoplasmic Translation, Cytoplasmic Ribosome biogenesis, Mitochondrial Ribosome, Mito Ribosome Assembly, ATP Hydrolysis + OXPHOS). Each dot is one biological replicate; colour = genotype (Ctrl grey, G32A blue, R403C vermillion). White rings = within-cell median; shaded bands = IQR; white lines connect D35 → D65 medians per genotype. The panel reveals what each contrast-level GSEA pattern label means at the replicate level (driver labels defined in Methods §e: `both_moving` = both arms move in opposing directions across timepoints; `ctrl_driven` = control descent closes the gap while mutants stay flat; `mutant_driven` and `neither_moving` are the symmetric and null cases). Synaptic Ribosome and Cytoplasmic Translation show a true sample-level crossover (`both_moving` Sign_reversal); Cytoplasmic Ribosome — biogenesis is contrast-level Compensation but replicate-level `ctrl_driven` — Ctrl medians descend from ≈ +0.4 toward ≈ −0.4 while mutants stay flat. shaded bands are IQR (25th–75th percentile of per-replicate GSVA scores within each genotype × day cell)

Supplementary Figure S8 | Semantic Pathway Overview. Rows: individual pathways, stacked in blocks by semantic category (pathways required to reach FDR < 0.05 in at least one of the nine GSEA contrasts). Columns: Early, TrajDev, Late × mutation (G32A | R403C). Dot color encodes NES on a blue–white–orange diverging scale clipped at ±3.5; dot size encodes $-\log_{10}$(BH-FDR) with reference markers at FDR = 0.001 / 0.01 / 0.05 / 0.1; black dot outline border = FDR < 0.05, grey otherwise. Of 73 mitochondrial pathways significantly enriched at any stage (identified by "mitochondri", "OXPHOS", "respiratory", "electron transport" or "ATP synthase" across all databases plus MitoCarta origin), 23 of 27 G32A active responders (85.2%) and 13 of 32 R403C (40.6%) carry the Compensation pattern label; active responders are pathways with TrajDev FDR < 0.05 and |NES_TrajDev| > 0.5.

Supplementary Figure S9 | 35 and 65 DIV changes in PSD95:Synaptophysin overlap via rotation. (A) Spinning disk confocal max intensity projection of 35 DIV cortical neurons from biological replicates (n=3) of control, G32A, and R403C using the 100x objective; scale, 5 μm, with the PSD95 channel rotated 90, and 180degrees. (B) Quantification of the mean PSD95:Synaptophysin overlap of a 90-degree rotation of PSD95 and 180-degree rotation of PSD95(n=3; one-way ANOVA with Dunnett’s post-hoc analysis; 90-degree, F(2, 6) = 2.349, G32A p= 0.4190, R403C p= 0.1255; 180-degree, F(2, 6) = 1.497, G32A p= 0.9355, R403C p= 0.2537;, Rout’s Outliers test, Q=1%, R403C outlier = 11.7). Error bars represent mean ± SEM.

(C) Spinning disk confocal max intensity projection of 65 DIV cortical neurons from biological replicates (n=3) of control, G32A, and R403C using the 100x objective; scale, 5 μm, with the PSD95 channel rotated 90 and 180degrees. (D) Quantification of the mean PSD95:Synaptophysin overlap of a 90-degree rotation of PSD95 (left panel), and of a 180-degree rotation of PSD95(n=3; one-way ANOVA with Dunnett’s post-hoc analysis; 90-degree, F(2, 6) = 0.3365, G32A p= 0.7005, R403C p= 0.9984, Rout’s Outliers test, Q=1%, G32A outliers = 12.64, 12.55, 12.62; 180-degree, F(2, 6) = 0.04052, G32A p= 0.9909, R403C p= 0.9439, Rout’s Outliers

test, Q=1%, control outliers = 11.88, 13.82 and R403C outliers = 15.15,14.73Error bars represent mean ± SEM.

Supplementary Figure S10 | Protein Expression Analysis. (A) Western blots of PNPO (left panel), PCDHGC3 (center panel), and NNAT (right panel) in G32A and R403C cultures 100+ DIV (D) Quantification of western blots of PNPO (left panel), PCDHGC3 (center panel), and NNAT (right panel) (n=3 blots; one-way ANOVA; PNPO F2,6=9.06, df=0.73, G32A p=0.026, R403C p=0.80; PCDHGC3 F2,6=12.99, df=0.55, G32A p=0.0067, R403C p=0.90; NNAT F2,6=10.98, df=2.07, G32A p=0.01, R403C p=0.01). Error bars represent mean ± SEM.

Supplementary Figure S11 | Basal calcium signaling levels. (A) Spinning disk confocal max intensity projection of 200+ DIV cortical neurons from biological replicates (n=3) of control and R403C. Neurons were labeled with 5 uM Cal-520 AM for 60 min at 37 °C. Live-cell imaging was performed using spinning disk confocal microscopy, with images acquired every 10 s for 5 min using the 100x objective; scale, 40 µm (DAPI = magenta and Cal-520AM = green blue fire). (B) The mean peak relative fluorescence (dF/F_0) of Cal-520AM with a baseline of the 8th percentile for normalization (n=3; unpaired t-test with Welch's correction; t=14.82, df=3.996, p= 0.0001). (C) Graph of the relative mean fluorescent intensity of Cal-520AM plotted against time (left panel), and quantification of the relative mean fluorescent intensity of Cal-520AM (right panel) (n=3; unpaired t-test with Welch's correction; t=3.816, df=4.000, p= 0.0188). (D) Graph of the relative mean absolute deviation of Cal-520AM fluorescent intensity plotted against time (left panel), and quantification of the relative mean absolute deviation of Cal-520AM fluorescent intensity (right panel) (n=3; unpaired t-test with Welch's correction; t=4.247, df=2.053, p= 0.0489; Rout's Outliers test, Q=1%, control outliers = 3.703, 3.562; R403C outlier = 7.631, 7.921, 7.413). Error bars represent mean ± SEM. Relative values were background corrected and normalized to the mean of controls at time 0.

Supplementary Figure S12 | Geometric (NES_Late, NES_Early) scatter. Geometric interpretation of trajectory-pattern classification . Each point is one of up to 5,267 ever-significantly enriched pathways (per-mutation n given in panel titles after excluding pathways with missing NES), plotted in (NES_Late, NES_Early) coordinates — x = D65 mutant-vs-Ctrl NES (developmental outcome), y = D35 mutant-vs-Ctrl NES (initial state). Color encodes super-category from `01_Scripts/Python/pattern_definitions.py` (Active_Compensation, Active_Reversal, Active_Progression, Passive, Late_onset, Other); the colour axis disambiguates patterns that occupy the same (NES_Late, NES_Early) quadrant but differ on whether TrajDev reached FDR < 0.05 with |NES_TrajDev| > 0.5. Dotted diagonal: NES_Late = NES_Early (no trajectory change). Dashed axis lines at NES = 0; quadrant labels indicate pattern regions. Top pathways labeled by priority score (MitoCarta, SynGO, GO:BP, GO:CC prioritised; ties broken by |NES_TrajDev|).

TABLES

Supplementary Table S1 | Per-contrast differential-expression results. Per-contrast limma-voom DE tables for all 9 model contrasts × 21,122 detected genes Columns: gene symbol, logFC, AveExpr, t, P.Value, p.adj (BH)- logFC: Log_2 fold change (positive = upregulated in numerator condition; negative = downregulated)

- AveExpr: Average log_2 counts per million (CPM) across all samples

- t: Moderated t-statistic from empirical Bayes analysis

- P.Value: Raw p-value from moderated t-test

- adj.P.Val: False discovery rate (FDR)-adjusted p-value via Benjamini-Hochberg correction

- B: Log-odds of differential expression (B-statistic)

Genes are sorted by t-statistic (most upregulated to most downregulated in each contrast). Statistical method: Linear model fit via limma-voom with precision weights, TMM normalization, empirical Bayes moderation of standard errors, FDR correction via Benjamini-Hochberg method. Tables included are

- **Mutation effects at two developmental stages**

- **Panel A (`G32A_vs_Ctrl_D35`):** G32A mutation at 35 DIV (early maturation).

- **Panel B (`R403C_vs_Ctrl_D35`)**: R403C mutation at 35 DIV.

- **Panel C (`G32A_vs_Ctrl_D65`)**: G32A mutation at 65 DIV (mature stage).

- **Panel D (`R403C_vs_Ctrl_D65`)**: R403C mutation at 65 DIV.

Panels E-G: Maturation effects within each genotype (D65 vs D35)

- Panel E (`Time_Ctrl`): Control neuron maturation.

- Panel F (`Time_G32A`): G32A neuron maturation.

- Panel G (`Time_R403C`): R403C neuron maturation.

Panels H-I: Mutation-specific maturation effects (trajectory deviation contrasts)

- Panel H (`Maturation_G32A_specific`): G32A-specific maturation dynamics, calculated as `(Time_G32A) - (Time_Ctrl)` to isolate mutation-driven trajectory deviations. This is the TrajDev component of the trajectory framework (Early → TrajDev → Late).

- Panel I (`Maturation_R403C_specific`): R403C-specific maturation dynamics, calculated as `(Time_R403C) - (Time_Ctrl)`. Interpretation identical to Panel H.

Supplementary Table S2 | Maturation DEGs. Full union of the two within-mutation maturation DEG sets at FDR < 0.05: 154 genes partitioned by a `Membership` column into shared (n = 38; DE in both `Time_G32A` and `Time_R403C`), G32A_only (n = 30), and R403C_only (n = 86). Each row carries `G32A_logFC` / `G32A_FDR` / `G32A_dir` and `R403C_logFC` / `R403C_FDR` / `R403C_dir` regardless of which side passed the significance threshold, plus `Mean_logFC`. Sort: by `Membership` (shared, G32A_only, R403C_only), then by `Mean_logFC` descending within each block. Underwrites the Fig. 4D Euler set sizes and the "core of 38 genes (56% of Time_G32A; 31% of Time_R403C)"

Supplementary Table S3 | Master GSEA pathway enrichment table with trajectory pattern classifications. Canonical GSEA results across all 12 databases × 9 contrasts. Columns include NES, p.adjust (BH), core_enrichment (leading-edge genes), contrast, pattern label, per-mutation trajectory metrics.

Canonical GSEA results across all 12 databases × 9 contrasts. Columns include NES, p.adjust (BH), core_enrichment (leading-edge genes), contrast, pattern label, per-mutation trajectory metrics. This master table integrates:

1. GSEA statistics for 9 contrasts (Early/Late mutation effects, TrajDev, Time effects) × >12,000 pathways = 109,989 pathway-contrast combinations

2. Trajectory framework mapping: NES and FDR values for Early (D35 effects), TrajDev (mutation-specific maturation), Late (D65 effects), and Time contrasts for both mutations

3. Pattern classifications: Each pathway assigned to one of 8 mutually exclusive patterns (Compensation, Sign reversal, Progressive, Natural improvement, Natural worsening, Late onset, Transient, Complex) for both G32A and R403C

4. Confidence levels: High/Medium confidence for pattern assignments based on statistical thresholds (FDR < 0.05 = High, FDR < 0.10 = Medium)

5. Super-category groupings: Simplified pattern groupings (Active_Compensation, Active_Reversal, Passive, Late_onset, Other) for high-level analysis

Column descriptions:

- pathway_id: Unique pathway identifier (database::ID)
- database: Source database (MitoCarta, SynGO, Hallmark, KEGG, Reactome, GO:BP/CC/MF, CGP, Canonical, TF, WikiPathways)
- ID, Description: Pathway identifiers and names
- contrast: Specific contrast being tested (e.g., G32A_vs_Ctrl_D35, Maturation_G32A_specific)
- new_name: Trajectory stage label (Early_G32A, TrajDev_G32A, Late_G32A, etc.)
- category: Trajectory component (Early, TrajDev, Late, Time)
- mutation: G32A or R403C
- NES: Normalized enrichment score (positive = upregulated, negative = downregulated)
- pvalue, p.adjust, qvalue: Statistical significance values
- enrichmentScore: Raw enrichment score from GSEA
- setSize: Number of genes in pathway after filtering
- Pattern_G32A, Pattern_R403C: Trajectory pattern classification (8 patterns; see Methods)
- Confidence_G32A, Confidence_R403C: High (FDR < 0.05 for key stages) or Medium (0.05 ≤ FDR < 0.10)

- Super_Category_G32A, Super_Category_R403C: Simplified pattern grouping (5 categories)
- change_consistency: Relationship between mutations (Consistent, Inconsistent_G32A-more-severe, Inconsistent_R403C-more-severe, Opposite_directions)
- NES_Early_G32A ... NES_Time_R403C: NES values for all trajectory stages (Early, TrajDev, Late) and Time contrasts for both mutations
- p.adjust_Early_G32A ... p.adjust_Time_R403C: FDR-adjusted p-values for all trajectory stages and Time contrasts
- ever_significant: TRUE if pathway reaches FDR < 0.05 in any contrast
- ever_significant_trajectory: TRUE if pathway reaches FDR < 0.05 in any trajectory stage (Early/TrajDev/Late)

Pattern definitions:

1. Compensation: Early defect (FDR < 0.05, |NES| > 0.5) + TrajDev opposes Early (FDR < 0.05, |NES| > 0.5, opposite sign) + Late improved ≥30% from Early → Active homeostatic response
2. Sign_reversal: Early and Late have opposite signs + TrajDev opposes Early (FDR < 0.05, |NES| > 0.5) → Trajectory crosses zero (e.g., synaptic ribosome pathways: upregulated D35 → downregulated D65)
3. Progressive: Early defect + TrajDev amplifies defect (same sign, FDR < 0.05) + Late worsened ≥30% → Deterioration during maturation
4. Natural_improvement: Early defect + TrajDev not significant + Late improved ≥30% → Passive recovery without active compensation
5. Natural_worsening: Early defect + TrajDev not significant + Late worsened ≥30% → Passive deterioration
6. Late_onset: No significant Early defect (FDR ≥ 0.10) + Late defect emerges (FDR < 0.10) → Delayed pathology

7. Transient: Strong Early defect (FDR < 0.05, |NES| > 1.0) + Late resolved (FDR ≥ 0.10, |NES| < 0.5) → Early stress resolves
8. Complex: Does not fit above patterns (multiphasic, inconsistent, or insufficient effect sizes)

Thresholds:

- Significance: FDR < 0.05 (High confidence), FDR < 0.10 (Medium confidence)
- Effect size: |NES| > 0.5 (minimum), |NES| > 1.0 (strong)
- Improvement/worsening: |Late|/|Early| < 0.7 (≥30% reduction) or > 1.3 (≥30% increase)

Supplementary Table S4 | Sensitivity Results. Pattern-classification counts and percentages for every (threshold combination × mutation) on the **12,221-pathway full background** (every pathway tested by GSEA, classified or not) [`Sensitivity_Analysis/sensitivity_results.csv`, 162 rows = 81 combinations × 2 mutations]. Columns: `combination_id`, the four threshold values (`NES_EFFECT`, `NES_STRONG`, `IMPROVEMENT_RATIO`, `WORSENING_RATIO`), `mutation`, `n_pathways = 12,221`, raw counts and percentages for each pattern (`Compensation` / `Compensation_pct`, `Sign_reversal` / `_pct`, `Progressive` / `_pct`, `Natural_improvement`, `Natural_worsening`, `Passive_total` / `Passive_pct`, `Late_onset` / `_pct`, `Transient` / `_pct`, `Complex` / `_pct`), `Insufficient_data`, and a boolean `is_default` flag marking the default-threshold row (0.5 / 1.0 / 0.7 / 1.3). Source for Supp. Fig. (Threshold-robustness sensitivity analysis**) Panels A and B**. Threshold semantics defined in the **Threshold parameters** block above.

Supplementary Table S5 | sensitivity_5267universe. Pattern-classification counts for every (threshold combination × mutation) restricted to the **5,267 ever-significantly enriched universe** — the RESULTS-section denominator (FDR < 0.05 in any of nine GSEA contrasts) [`**Supplementary/sensitivity_5267universe.csv**`, 162 rows = 81 combinations × 2 mutations].

Columns use the abbreviated threshold codes — see **Threshold parameters** block above for full names: `ne` (`NES_EFFECT`), `ns` (`NES_STRONG`), `ir` (`IMPROVEMENT_RATIO`), `wr` (`WORSENING_RATIO`); `mut` (mutation), `n_total = 5267`, `Comp` (Compensation), `SR` (Sign_reversal), `Prog` (Progressive), `NI` (Natural_improvement), `NW` (Natural_worsening), `LO` (Late_onset), `Tr` (Transient), `Complex` (counts); `Classif` = classifiable subset = `n_total − Complex − Insufficient_data`; `Comp_class_pct = Comp / Classif × 100` (Compensation as % of classifiable); `NI_class_pct = NI / Classif × 100`.

Supplementary Table S6 | Per-database pattern. Pathway-pattern counts and proportions per database. Underwrites the per-database pattern heatmap supplementary figure.

Supplementary Table S7 | Per-replicate GSVA scores (long format). Per-replicate GSVA enrichment scores across all classifiable pathways in long format, with per-pathway GSVA driver labels (`mutant_driven`, `ctrl_driven`, `both_moving`, `neither_moving`). Supports the replicate-level GSVA supplementary figure and powers the interactive dashboard

Supplementary Table S8 | Ribosome compartment summary. Per-pathway compartment annotation and trajectory metrics for the cytoplasmic / synaptic / mitochondrial ribosome universe (n = 95). Columns: pathway ID, compartment, Jaccard with SynGO synaptic-ribosome set, per-stage NES and FDR for both mutations, pattern label.

Supplementary Table S9 | Threshold-sensitivity digest. 81-combination threshold-sensitivity classification on the 5,267 universe; full per-pathway × per-threshold-combo file sensitivity_5267universe.csv

Supplementary Table S10 | Claim Stability. Stability of key compensation claims across an 81-combination NES-threshold sweep. Robustness of the GSEA-based pathway classifier was assessed by re-running the classifier over a four-dimensional grid of decision thresholds — effect-size cut-off (NES_EFFECT ∈ {0.4, 0.5, 0.6}), strong-effect cut-off (NES_STRONG ∈ {0.8, 1.0, 1.2}), rescue-ratio cut-off (IMPROVEMENT_RATIO ∈ {0.6, 0.7, 0.8}) and worsening-ratio cut-off (WORSENING_RATIO ∈ {1.25, 1.30, 1.40}) — yielding 81 threshold combinations per mutant. Each row corresponds to one combination and reports, separately for the G32A and R403C mutants: (i) the number and percentage of pathways classified as compensatory (comp_count_, comp_pct_); (ii) the number of pathways receiving any non-neutral classification (classifiable_) together with the fraction of that denominator accounted for by compensation (comp_of_classifiable_); and (iii) the percentage of pathways classified as progressive-only-rare (prog_pct_). Five Boolean indicators summarise whether each combination supports the manuscript's headline claims: compensation is the dominant pattern in the classifiable pool of each mutant (comp_dominates_classifiable_); R403C exhibits greater compensation than G32A (R403C_more_compensation; raw gap, compensation_diff); the progressive pattern remains rare (<1% of classifiable; progressive_rare_\); and the compensatory fraction exceeds the passive (untreated) baseline in each mutant (comp_exceeds_passive_). The threshold combination used in the main text is flagged by is_default = True.
