# Supplementary material for: DRP1 mutations associated with EMPF1 encephalopathy perturb the transcriptional profile and maturation of cortical neurons
Source: J Neurodev Disord. 2026 Jun 26;18:52. doi: 10.1186/s11689-026-09713-0 (PMC13563865; doi:10.1186/s11689-026-09713-0)

Supplementary Figure S1

A

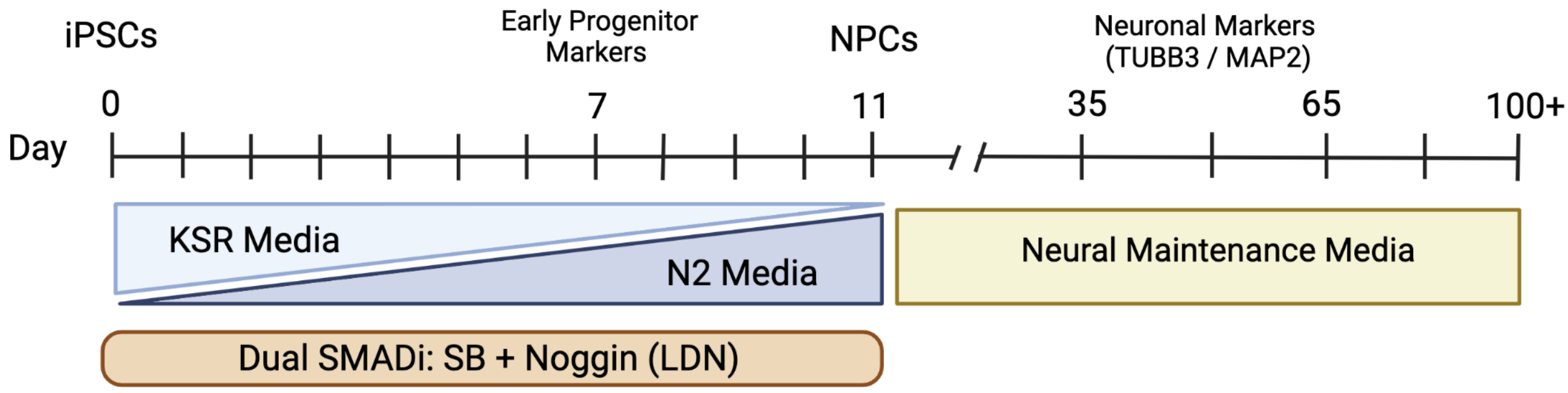

B

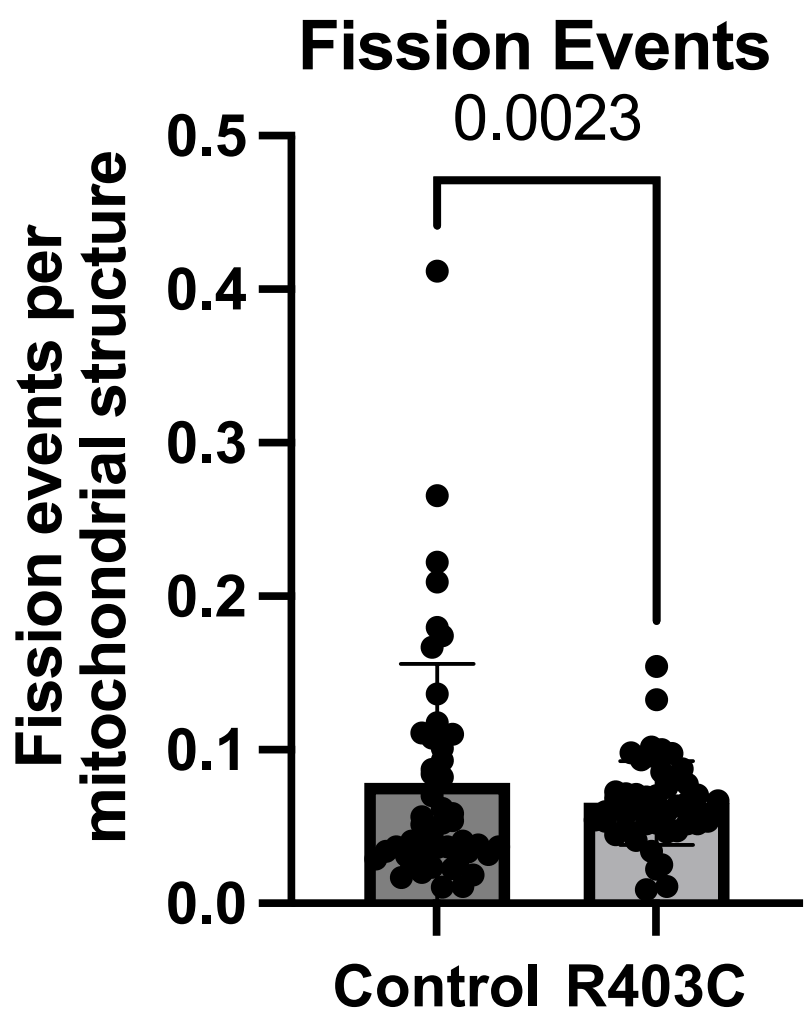

C

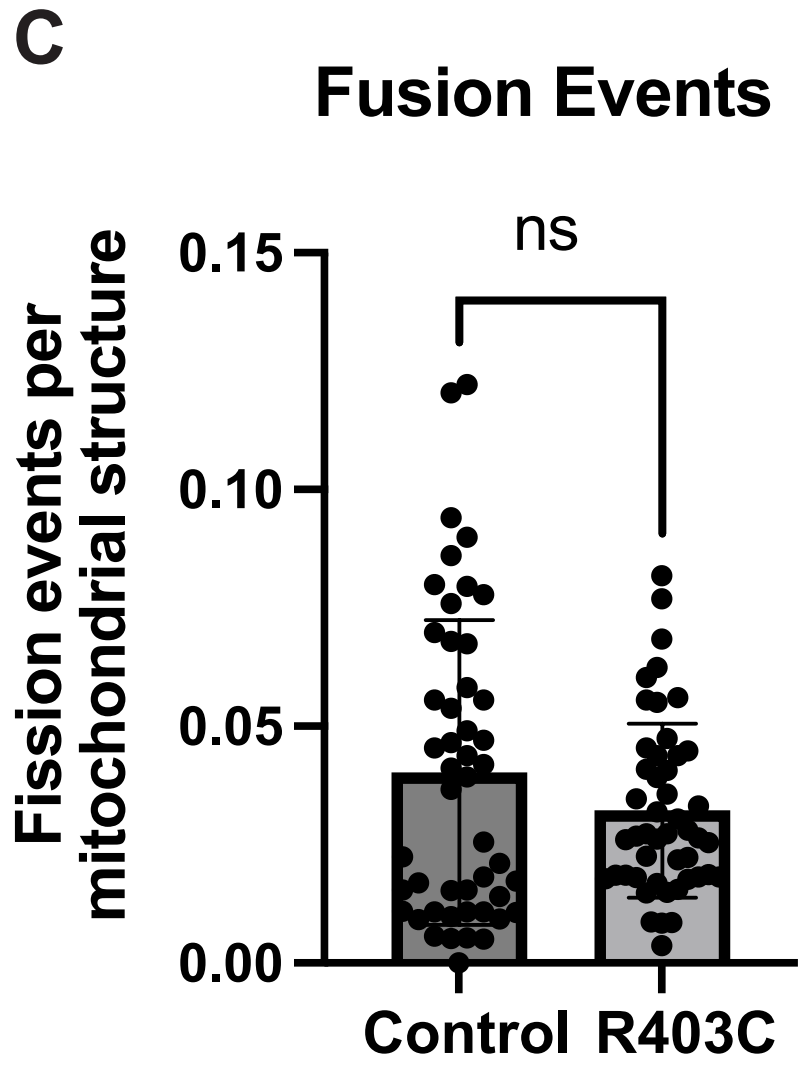

Supplement: Supplementary file 5 [file 11689_2026_9713_MOESM1_ESM.pdf]
