# Supplementary material for: DRP1 mutations associated with EMPF1 encephalopathy perturb the transcriptional profile and maturation of cortical neurons
Source: J Neurodev Disord. 2026 Jun 26;18:52. doi: 10.1186/s11689-026-09713-0 (PMC13563865; doi:10.1186/s11689-026-09713-0)

Supplementary Figure S5

Per-databasetrajectory-pattern frequencies (ever-significant pathways, FDR<0.05 at any stage in either mutation)

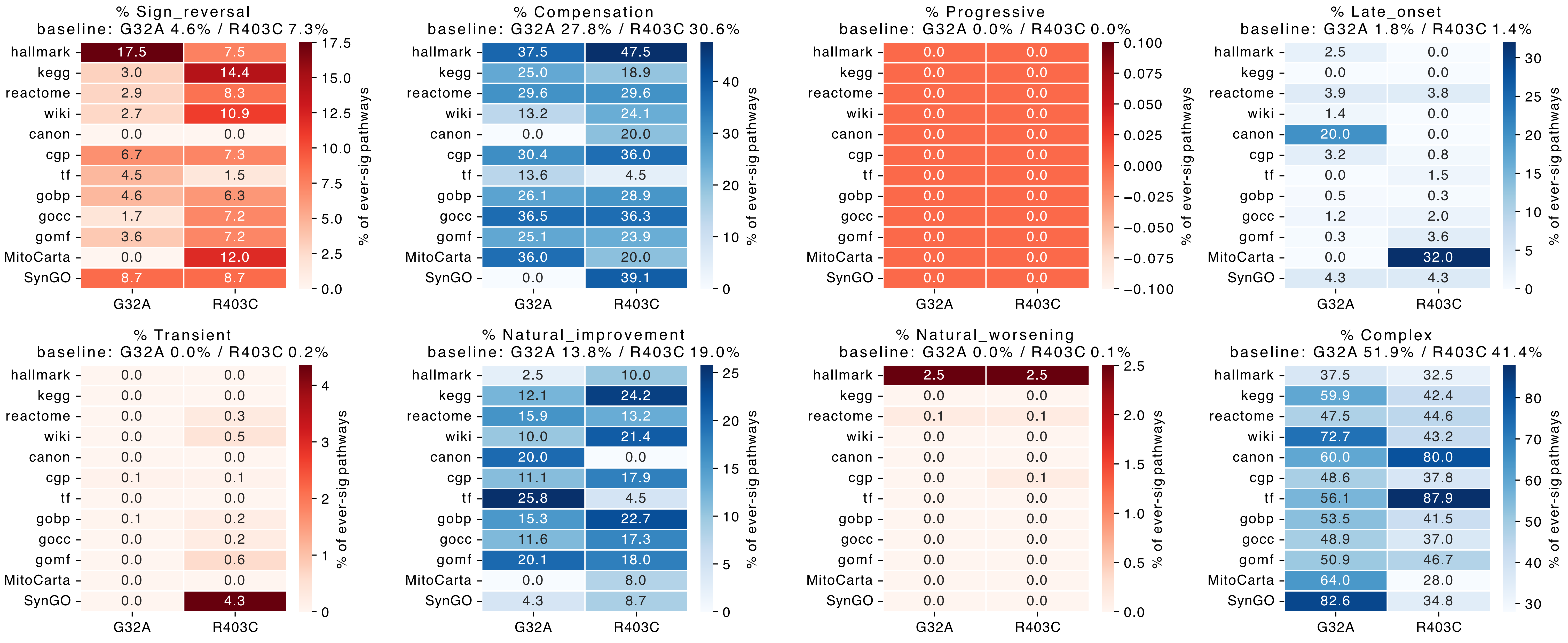

Supplement: Supplementary file 9 [file 11689_2026_9713_MOESM5_ESM.pdf]
