# Supplementary material for: DRP1 mutations associated with EMPF1 encephalopathy perturb the transcriptional profile and maturation of cortical neurons
Source: J Neurodev Disord. 2026 Jun 26;18:52. doi: 10.1186/s11689-026-09713-0 (PMC13563865; doi:10.1186/s11689-026-09713-0)
Supplement: Supplementary file 11 [file 11689_2026_9713_MOESM7_ESM.pdf]

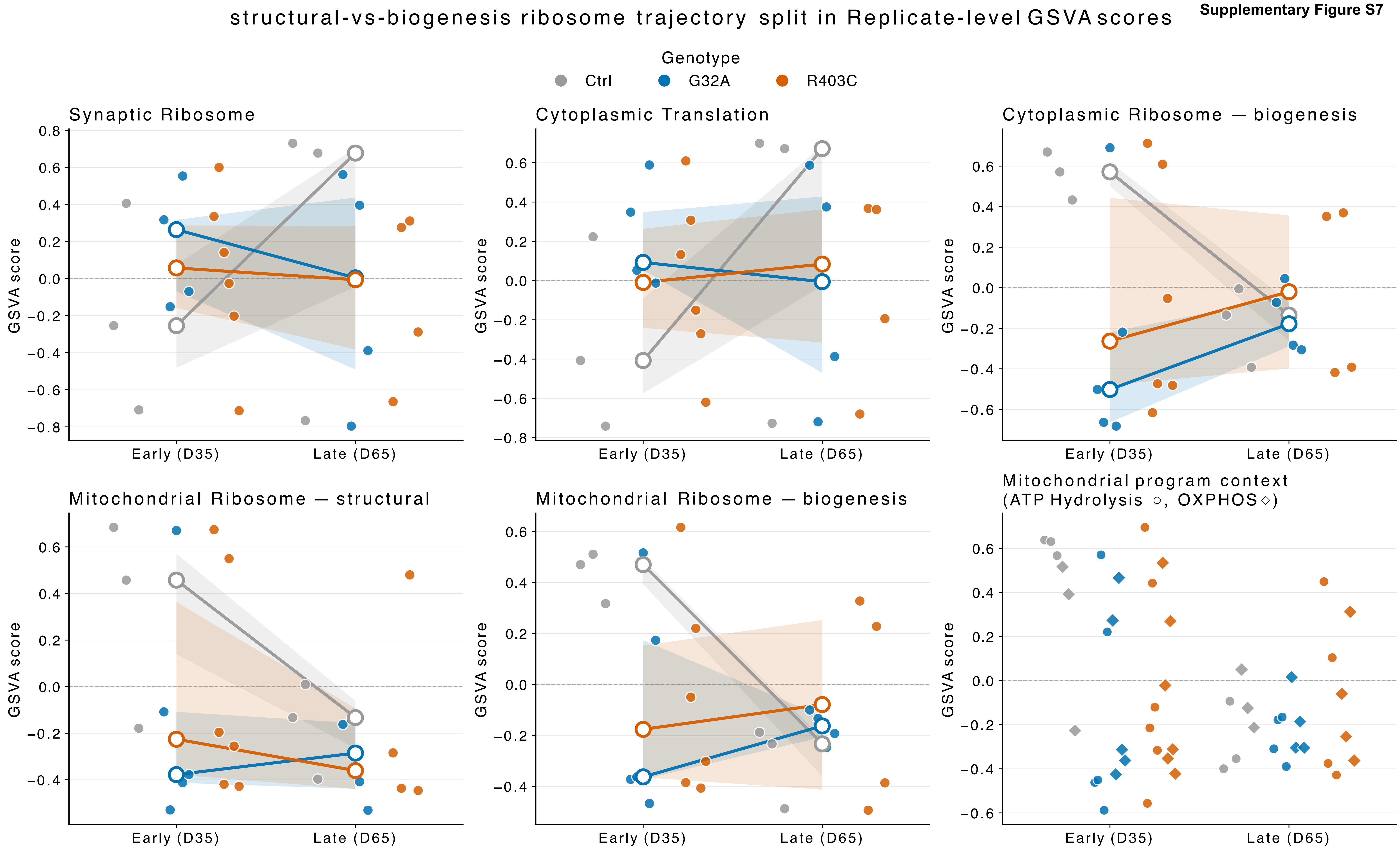

structural-vs-biogenesis ribosome trajectory split in Replicate-level GSVA scores
Genotype
Ctrl
G32A
R403C
Synaptic Ribosome
Cytoplasmic Translation
Cytoplasmic Ribosome — biogenesis
Mitochondrial Ribosome — structural
Mitochondrial Ribosome — biogenesis
Mitochondrial program context
(ATP Hydrolysis ○, OXPHOS ◇)
GSVA score
Early (D35)
Late (D65)
0.8
0.6
0.4
0.2
0.0
−0.2
−0.4
−0.6
−0.8
