# Supplementary material for: DRP1 mutations associated with EMPF1 encephalopathy perturb the transcriptional profile and maturation of cortical neurons
Source: J Neurodev Disord. 2026 Jun 26;18:52. doi: 10.1186/s11689-026-09713-0 (PMC13563865; doi:10.1186/s11689-026-09713-0)
Supplement: Supplementary file 12 [file 11689_2026_9713_MOESM8_ESM.pdf]

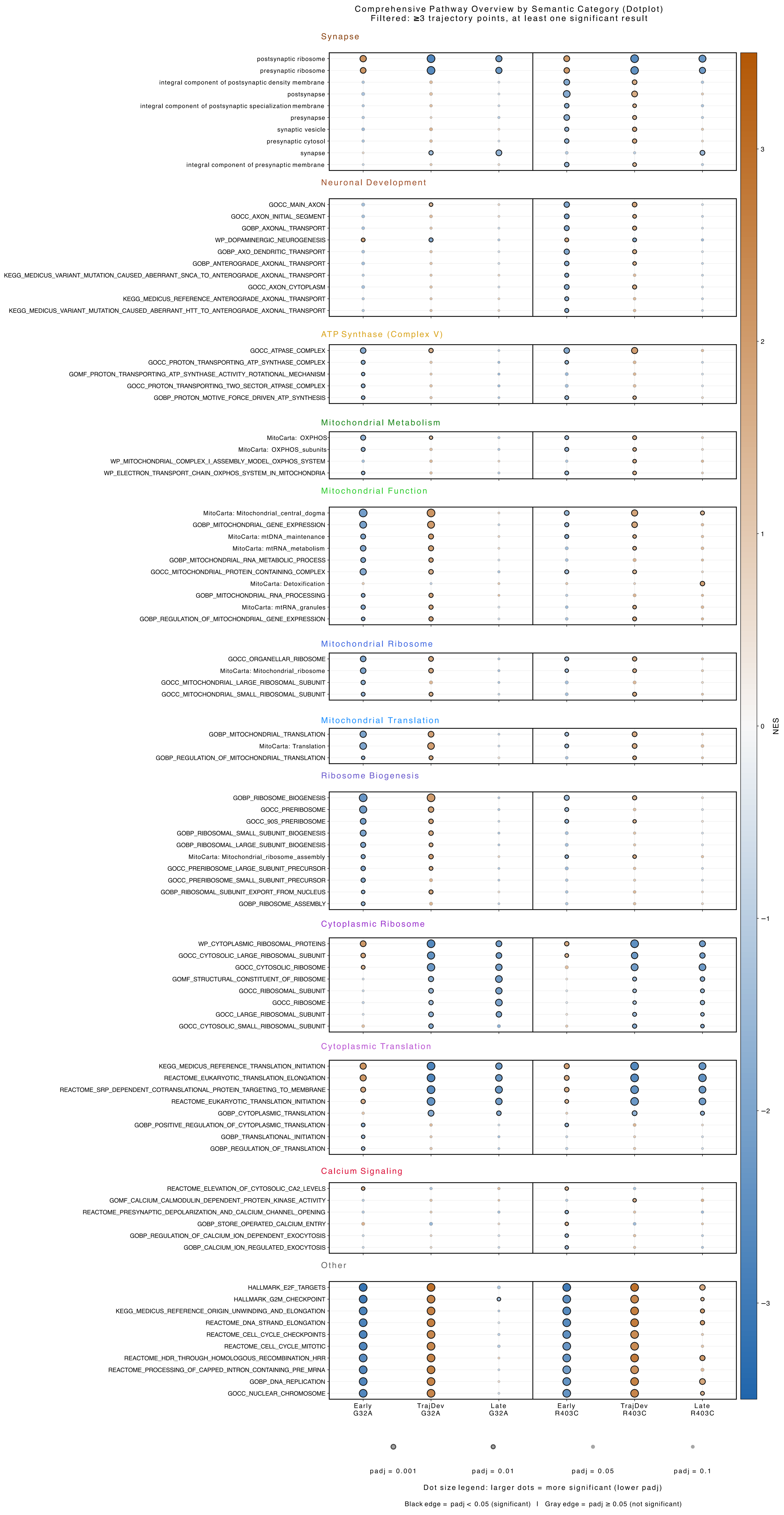
Comprehensive Pathway Overview by Semantic Category (Dotplot)
Filtered: ≥3 trajectory points, at least one significant result
Synapse
postsynaptic ribosome
presynaptic ribosome
integral component of postsynaptic density membrane
postsynapse
integral component of postsynaptic specialization membrane
presynapse
synaptic vesicle
presynaptic cytosol
synapse
integral component of presynaptic membrane
Neuronal Development
GOCC_MAIN_AXON
GOCC_AXON_INITIAL_SEGMENT
GOBP_AXONAL_TRANSPORT
WP_DOPAMINERGIC_NEUROGENESIS
GOBP_AXO_DENDRITIC_TRANSPORT
GOBP_ANTEROGRADE_AXONAL_TRANSPORT
KEGG_MEDICUS_VARIANT_MUTATION_CAUSED_ABERRANT_SNCA_TO_ANTEROGRADE_AXONAL_TRANSPORT
GOCC_AXON_CYTOPLASM
KEGG_MEDICUS_REFERENCE_ANTEROGRADE_AXONAL_TRANSPORT
KEGG_MEDICUS_VARIANT_MUTATION_CAUSED_ABERRANT_HTT_TO_ANTEROGRADE_AXONAL_TRANSPORT
ATP Synthase (Complex V)
GOCC_ATPASE_COMPLEX
GOCC_PROTON_TRANSPORTING_ATP_SYNTHASE_COMPLEX
GOMF_PROTON_TRANSPORTING_ATP_SYNTHASE_ACTIVITY_ROTATIONAL_MECHANISM
GOCC_PROTON_TRANSPORTING_TWO_SECTOR_ATPASE_COMPLEX
GOBP_PROTON_MOTIVE_FORCE_DRIVEN_ATP_SYNTHESIS
Mitochondrial Metabolism
MitoCarta: OXPHOS
MitoCarta: OXPHOS_subunits
WP_MITOCHONDRIAL_COMPLEX_I_ASSEMBLY_MODEL_OXPHOS_SYSTEM
WP_ELECTRON_TRANSPORT_CHAIN_OXPHOS_SYSTEM_IN_MITOCHONDRIA
Mitochondrial Function
MitoCarta: Mitochondrial_central_dogma
GOBP_MITOCHONDRIAL_GENE_EXPRESSION
MitoCarta: mtDNA_maintenance
MitoCarta: mtRNA_metabolism
GOBP_MITOCHONDRIAL_RNA_METABOLIC_PROCESS
GOCC_MITOCHONDRIAL_PROTEIN_CONTAINING_COMPLEX
MitoCarta: Detoxification
GOBP_MITOCHONDRIAL_RNA_PROCESSING
MitoCarta: mtRNA_granules
GOBP_REGULATION_OF_MITOCHONDRIAL_GENE_EXPRESSION
Mitochondrial Ribosome
GOCC_ORGANELLAR_RIBOSOME
MitoCarta: Mitochondrial_ribosome
GOCC_MITOCHONDRIAL_LARGE_RIBOSOMAL_SUBUNIT
GOCC_MITOCHONDRIAL_SMALL_RIBOSOMAL_SUBUNIT
Mitochondrial Translation
GOBP_MITOCHONDRIAL_TRANSLATION
MitoCarta: Translation
GOBP_REGULATION_OF_MITOCHONDRIAL_TRANSLATION
Ribosome Biogenesis
GOBP_RIBOSOME_BIOGENESIS
GOCC_PRERIBOSOME
GOCC_90S_PRERIBOSOME
GOBP_RIBOSOMAL_SMALL_SUBUNIT_BIOGENESIS
GOBP_RIBOSOMAL_LARGE_SUBUNIT_BIOGENESIS
MitoCarta: Mitochondrial_ribosome_assembly
GOCC_PRERIBOSOME_LARGE_SUBUNIT_PRECURSOR
GOCC_PRERIBOSOME_SMALL_SUBUNIT_PRECURSOR
GOBP_RIBOSOMAL_SUBUNIT_EXPORT_FROM_NUCLEUS
GOBP_RIBOSOME_ASSEMBLY
Cytoplasmic Ribosome
WP_CYTOPLASMIC_RIBOSOMAL_PROTEINS
GOCC_CYTOSOLIC_LARGE_RIBOSOMAL_SUBUNIT
GOCC_CYTOSOLIC_RIBOSOME
GOMF_STRUCTURAL_CONSTITUENT_OF_RIBOSOME
GOCC_RIBOSOMAL_SUBUNIT
GOCC_RIBOSOME
GOCC_LARGE_RIBOSOMAL_SUBUNIT
GOCC_CYTOSOLIC_SMALL_RIBOSOMAL_SUBUNIT
Cytoplasmic Translation
KEGG_MEDICUS_REFERENCE_TRANSLATION_INITIATION
REACTOME_EUKARYOTIC_TRANSLATION_ELONGATION
REACTOME_SRP_DEPENDENT_COTRANSLATIONAL_PROTEIN_TARGETING_TO_MEMBRANE
REACTOME_EUKARYOTIC_TRANSLATION_INITIATION
GOBP_CYTOPLASMIC_TRANSLATION
GOBP_POSITIVE_REGULATION_OF_CYTOPLASMIC_TRANSLATION
GOBP_TRANSLATIONAL_INITIATION
GOBP_REGULATION_OF_TRANSLATION
Calcium Signaling
REACTOME_ELEVATION_OF_CYTOSOLIC_CA2_LEVELS
GOMF_CALCIUM_CALMODULIN_DEPENDENT_PROTEIN_KINASE_ACTIVITY
REACTOME_PRESYNAPTIC_DEPOLARIZATION_AND_CALCIUM_CHANNEL_OPENING
GOBP_STORE_OPERATED_CALCIUM_ENTRY
GOBP_REGULATION_OF_CALCIUM_ION_DEPENDENT_EXOCYTOSIS
GOBP_CALCIUM_ION_REGULATED_EXOCYTOSIS
Other
HALLMARK_E2F_TARGETS
HALLMARK_G2M_CHECKPOINT
KEGG_MEDICUS_REFERENCE_ORIGIN_UNWINDING_AND_ELONGATION
REACTOME_DNA_STRAND_ELONGATION
REACTOME_CELL_CYCLE_CHECKPOINTS
REACTOME_CELL_CYCLE_MITOTIC
REACTOME_HDR_THROUGH_HOMOLOGOUS_RECOMBINATION_HRR
REACTOME_PROCESSING_OF_CAPPED_INTRON_CONTAINING_PRE_MRNA
GOBP_DNA_REPLICATION
GOCC_NUCLEAR_CHROMOSOME
Early G32A
TrajDev G32A
Late G32A
Early R403C
TrajDev R403C
Late R403C
NES
3
2
1
0
−1
−2
−3
padj = 0.001
padj = 0.01
padj = 0.05
padj = 0.1
Dot size legend: larger dots = more significant (lower padj)
Black edge = padj < 0.05 (significant) | Gray edge = padj ≥ 0.05 (not significant)
